# Simultaneously boosting the conjugation, brightness and solubility of organic fluorophores by using AIEgens†

**DOI:** 10.1039/d0sc03423a

**Published:** 2020-07-27

**Authors:** Ji Qi, Xingchen Duan, Yuanjing Cai, Shaorui Jia, Chao Chen, Zheng Zhao, Ying Li, Hui-Qing Peng, Ryan T. K. Kwok, Jacky W. Y. Lam, Dan Ding, Ben Zhong Tang

**Affiliations:** Department of Chemistry, The Hong Kong Branch of Chinese National Engineering Research Centre for Tissue Restoration and Reconstruction, Institute for Advanced Study, Department of Chemical and Biological Engineering, Institute of Molecular Functional Materials, The Hong Kong University of Science and Technology Clear Water Bay Kowloon Hong Kong China tangbenz@ust.hk; State Key Laboratory of Medicinal Chemical Biology, Key Laboratory of Bioactive Materials, Ministry of Education and College of Life Sciences, Nankai University Tianjin 300071 China dingd@nankai.edu.cn; Beijing Advanced Innovation Centre for Soft Matter Science and Engineering, Beijing University of Chemical Technology Beijing 100029 China; HKUST-Shenzhen Research Institute No. 9 Yuexing First RD, South Area, Hi-tech Park, Nanshan Shenzhen 518057 China; NSFC Centre for Luminescence from Molecular Aggregates, SCUT-HKUST Joint Research Institute, State Key Laboratory of Luminescent Materials and Devices, South China University of Technology Guangzhou 510640 China

## Abstract

Organic near-infrared (NIR) emitters hold great promise for biomedical applications. Yet, most organic NIR fluorophores face the limitations of short emission wavelengths, low brightness, unsatisfactory processability, and the aggregation-caused quenching effect. Therefore, development of effective molecular design strategies to improve these important properties at the same time is a highly pursued topic, but very challenging. Herein, aggregation-induced emission luminogens (AIEgens) are employed as substituents to simultaneously extend the conjugation length, boost the fluorescence quantum yield, and increase the solubility of organic NIR fluorophores, being favourable for biological applications. A series of donor–acceptor type compounds with different substituent groups (*i.e.*, hydrogen, phenyl, and tetraphenylethene (TPE)) are synthesized and investigated. Compared to the other two analogs, **MTPE-TP3** with TPE substituents exhibits the reddest fluorescence, highest brightness, and best solubility. Both the conjugated structure and twisted conformation of TPE groups endow the resulting compounds with improved fluorescence properties and processability for biomedical applications. The *in vitro* and *in vivo* applications reveal that the NIR nanoparticles function as a potent probe for tumour imaging. This study would provide new insights into the development of efficient building blocks for improving the performance of organic NIR emitters.

## Abstract 2

AIEgens are exploited to simultaneously extend the conjugation, boost the brightness, and increase the solubility of organic near-infrared fluorophores, representing a new strategy for developing high-performance emitters for biomedical imaging.

## Graphical abstract

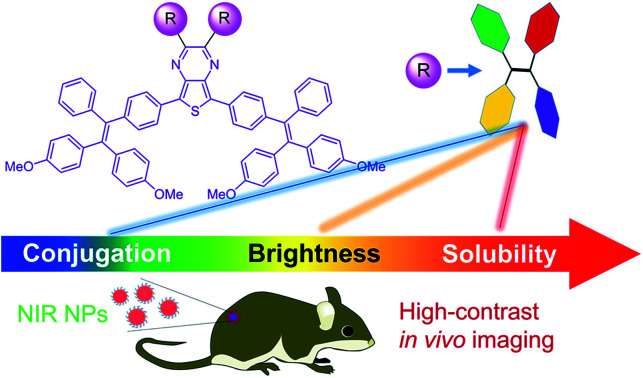

## Introduction

Near-infrared (NIR, >650 nm) fluorescence imaging has attracted considerable attention from both fundamental researchers and the clinical community, as it has lower autofluorescence interference, causes less photodamage, and has better penetration capability as compared with visible light, making it a preferable option for biological imaging.^1–3^ A lot of NIR fluorophores have been developed, for example, quantum dots, carbon nanomaterials, rare earth-doped nanoparticles (NPs), and organic emitters.^4–7^ Among them, organic materials hold great promise for clinical translation due to the salient merits of good biocompatibility, a well-defined chemical structure, facile modification, large-scale production, and potential degradation.^8–10^ Therefore, the development of efficient organic NIR emitters is highly important for advancing biomedical applications.^11,12^ At present, organic NIR molecules face several limitations, such as short emission wavelengths, low brightness, unsatisfactory processability, and the aggregation-caused quenching (ACQ) effect.^13–15^ Moreover, it is very difficult to optimize all these important performance at the same time. Organic NIR chromophores are usually developed by extending the conjugation length and using a donor–acceptor (D–A) approach, however, both would cause strong intermolecular π–π interactions, and give rise to more pronounced processability/solubility and ACQ issues.^16–18^ The most popularly used strategy is the introduction of a lot of alkyl chains to increase the solubility and disrupt the intermolecular interactions.^19,20^ Although effective, the bulky insulating groups would impact the photophysical properties adversely.^21,22^ As a result, the introduction of a large amount of alkyl chains is a compromised or temporary selection, which calls for more efficient methods.

After first being coined by Tang and co-workers in 2001, aggregation-induced emission (AIE) has been considered as an effective solution to the notorious ACQ phenomenon.^23,24^ AIE luminogens (AIEgens) are weak or non-luminescent in dilute solution but become highly emissive in the aggregate or solid state due to the restriction of intramolecular motion (RIM) mechanism.^25–27^ Freely rotating molecular rotors have been employed to endow the molecules with AIE signature, for example, hexphenylsilole (HPS) and tetraphenylethene (TPE).^28–31^ Many AIEgens with emission colours covering the entire visible spectral region and even the NIR range have been developed, and some of them exhibit great promise for biomedical applications.^32–35^ Nevertheless, the exploration of NIR AIEgens is suboptimal, and more studies are needed to optimize the emission wavelength and brightness simultaneously. One of the most serious obstacles for developing organic NIR luminogens is the low photoluminescence quantum yield (PLQY). According to the “energy gap law”, the brightness of organic molecules usually decreases as the emission wavelength red shifts, especially in the NIR region, because the large vibronic coupling between the ground and excited states, and the non-radiative deactivation pathways become dominant when the electronic bandgap decreases.^36–39^ Redshifting the emission wavelength and increasing the brightness at the same time are ideal for organic NIR bioprobes, but this is scarcely reported as it is indeed a challenging task.

In this contribution, we report the simultaneous bathochromic emission, boosted PLQY, and increased solubility of organic NIR fluorophores by simply introducing AIEgens as the substituent groups, which is beneficial for biological imaging. By employing the typical AIE building block, TPE, as the substituent unit, the absorption/emission wavelength red shifts with the extension of the conjugation length, the brightness increases greatly because of the suppressed non-radiative processes, and the solubility also increases since the molecular rotors disrupt the intermolecular interaction. Both *in vitro* and *in vivo* experiments suggest that the bioimaging performance is enhanced for the probe with AIEgen-based molecular rotors. This work for the first time demonstrates that AIEgens could significantly increase the conjugation, brightness and solubility of organic NIR luminogens, being better than the typically used alkyl chains, and represents a new strategy for developing high-performance fluorophores.

## Results and discussion

**Scheme 1:**
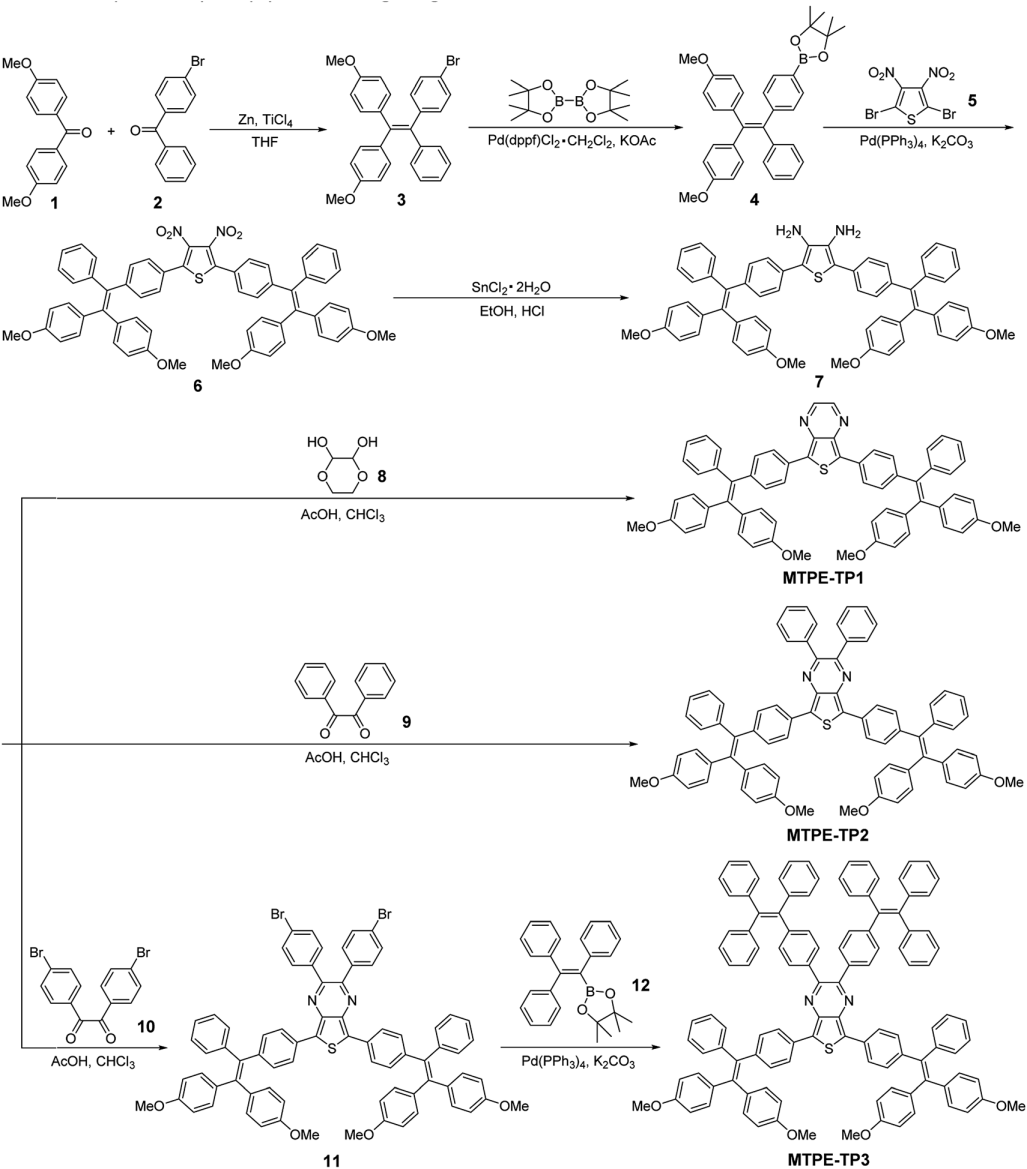
Synthetic route to **MTPE-TP1–3**.

The D–A structured molecules with methoxy-substituent TPE (MTPE) as the donor and thieno[3,4-*b*]pyrazine (TP) as the acceptor were synthesized, in which the electronic bandgaps could be tuned by changing the conjugation. TP is an electron-withdrawing group based on planar thiophene fused with pyrazine, which facilitates the intramolecular charge transfer (ICT) in D–A molecules. The MTPE unit exhibits stronger electron-donating properties than the widely used building block of AIEgen, TPE, and thus leading to a smaller bandgap and longer emission wavelength. Based on the molecular architecture of “MTPE–TP–MTPE”, a series of compounds with different substituents (*i.e.*, hydrogen, phenyl, and TPE) in the TP core (Scheme 1) were synthesized to study their influence on the molecular conjugation and conformation, and photophysical properties. The synthetic route of **MTPE-TP1–3** is presented in Scheme 1. The Suzuki coupling reaction was carried out between 2-(4-(2,2-bis(4-methoxyphenyl)-1-phenylvinyl)phenyl)-4,4,5,5-tetramethyl-1,3,2-dioxaborolane (**4**) and 2,5-dibromo-3,4-dinitrothiophene (**5**) to yield the dinitro molecule (**6**), followed by a reduction reaction to afford the diamine compound (**7**), which subsequently underwent cyclization with benzyl derivatives to obtain the TP structure. For **MTPE-TP3**, bromobenzene was first attached to the TP core, and then reacted with 4,4,5,5-tetramethyl-2-(1,2,2-triphenylvinyl)-1,3,2-dioxaborolane (**12**) to construct the TPE group. The intermediates and final compounds have been characterized using ^1^H NMR, ^13^C NMR and high-resolution mass spectra (HRMS). Detailed synthesis processes and characterization are presented in the ESI (Fig. S1–S22†).

**Fig. 1:**
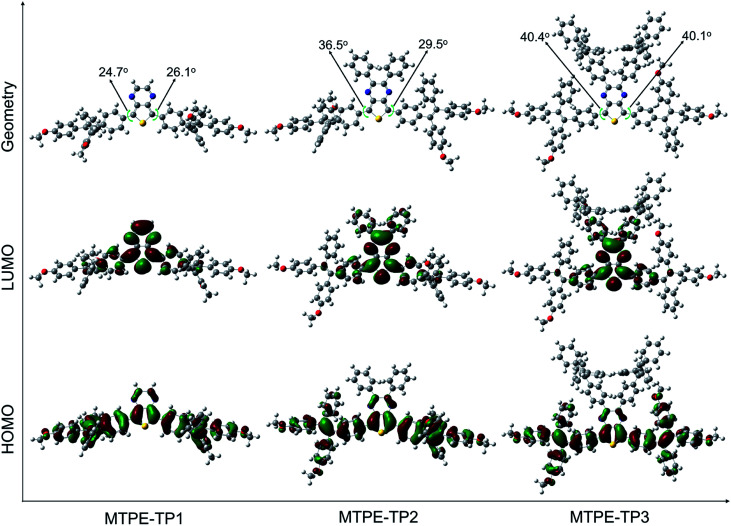
Optimized molecular geometries, and HOMO and LUMO distributions of **MTPE-TP1–3**.

To gain deeper insight into the molecular conformation, density functional theory (DFT) calculations were performed. As depicted in Fig. 1, **MTPE-TP1–3** possess rather twisted molecular geometries, in which the intramolecular rotors would dissipate the excited-state energy through the free high-frequency rotation of phenyl rings in solution, and the ACQ effect would be significantly inhibited in the aggregation/solid form, being favourable for realizing the AIE signature.^40,41^ Interestingly, it is obvious that these three molecules have different conjugations. For instance, the dihedral angles between TP and adjacent phenyl rings are 24.7°/26.1°, 36.5°/29.5°, and 40.4°/40.1° for **MTPE-TP1–3**, respectively. The increased backbone distortion is probably due to the steric resistance between MTPE and substituents in the pyrazine ring, which would impact the photophysical properties.^42,43^ The highest occupied molecular orbital (HOMO) is distributed in both MTPE and TP units (Fig. 1), suggesting good intramolecular conjugation. While the electron cloud of the lowest unoccupied molecular orbital (LUMO) is mainly located in the TP core, indicating an obvious intramolecular D–A interaction and ICT effect from MTPE to TP.^44,45^ It is noted that the electron density of the LUMOs of **MTPE-TP2,3** is also distributed in the phenyl rings attached to pyrazine, which is an indicator of better conjugation, and thus smaller bandgaps can be expected.

**Fig. 2:**
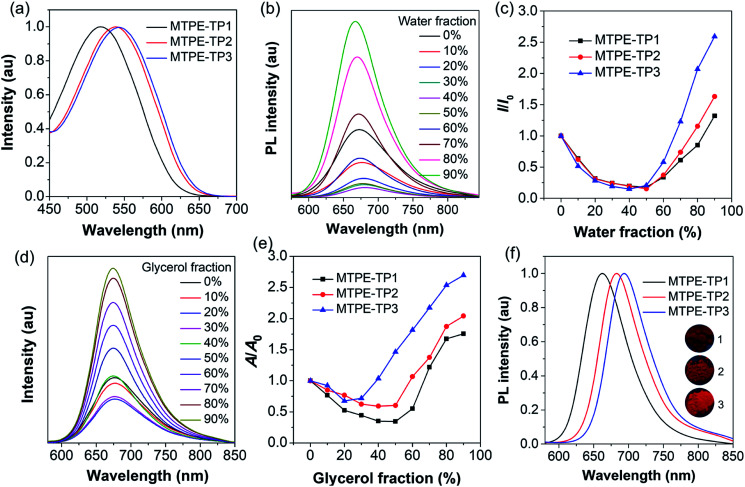
(a) Absorption spectra of **MTPE-TP1–3** in DMSO (10^−5^ M). (b) PL spectra of **MTPE-TP3** and (c) plot of the PL peak intensity *versus* water fraction (*f*_w_) in DMSO/water mixtures (10^−5^ M). *I*_0_ and *I* represent the PL intensities of the compounds in pure DMSO (*f*_w_ = 0) and DMSO/water mixtures with specific *f*_w_s, respectively. (d) PL spectra of **MTPE-TP3** and (e) plot of the PL peak intensity *versus* glycerol fraction (*f*_g_) in DMF/glycerol mixtures (10^−5^ M). *A*_0_ and *A* represent the PL intensities of the compounds in pure DMF and DMF/glycerol mixtures with specific *f*_g_s, respectively. (f) PL spectra of **MTPE-TP1–3** powders. The inset shows the photographs of powder emission under UV light (365 nm) irradiation.

We first evaluated the solubility, since processability is of vital importance for application of organic molecules. The solubilities of **MTPE-TP1–3** in dimethyl sulfoxide (DMSO) are 0.58, 0.36, and 1.22 mg mL^−1^, respectively. The lower solubility of **MTPE-TP2** as compared with **MTPE-TP1** can be ascribed to the extended conjugation, while the highest solubility of **MTPE-TP3** is likely due to the introduction of violently rotated TPE substituents. The absorption spectra of **MTPE-TP1–3** in DMSO (Fig. 2a) show the maximum absorption in sequence at 518 nm, 538 nm, and 543 nm, which are in good agreement with the calculation results that the conjugations of **MTPE-TP2,3** are better than that of **MTPE-TP1**, and the slightly longer absorption of **MTPE-TP3** is probably attributed to the contribution of conjugated TPE side groups. As shown in Fig. S23,† the photoluminescence (PL) spectra exhibit a similar trend to the absorption change, and **MTPE-TP3** shows the reddest emission. Of note, although the planarity/conjugation between MTPE and TP decreases from **MTPE-TP1** to **MTPE-TP3** for the increased dihedral angles, the introduction of phenyl and TPE substituents on TP contributes more to the molecular conjugation.

We next investigated the fluorescence properties by adding water (poor solvent) into DMSO solution (good solvent) of the compounds. The PL intensity decreases in low water fractions and then intensifies (Fig. 2b, c and S24†). The decrease of PL intensity and concurrent redshift of the emission wavelength can be explained by the solvatochromic effect in polar solvents (Fig. S25†), which is usually observed in D–A type compounds.^46,47^ The intensified PL intensity is due to the formation of aggregates, representing a typical AIE signature. The AIE amplitude increases from **MTPE-TP1** to **MTPE-TP3**, especially there is a notable enhancement for **MTPE-TP3**, which is likely due to the more twisted molecular geometry and inhibition of intermolecular interactions in the aggregate state. We further studied the PL properties in different viscosity environments, as molecular motions would be restricted in high viscosity.^48,49^ The PL spectra and corresponding intensity changes in dimethylformamide (DMF)/glycerol mixtures with various glycerol fractions are presented in Fig. 2d, e and S26.† When increasing the glycerol fractions, the PL intensity firstly decreases and then rises. This phenomenon could also be explained by the solvatochromism and AIE effect for the high polarity and viscosity of glycerol. In high glycerol fractions, the viscosity becomes rather high, and the molecular motions are restricted greatly, which could effectively suppress the energy consumption *via* non-radiative deactivation.^50,51^ The PL spectra of solid powders (Fig. 2f) suggest a bathochromic shift compared to solution states and very bright emission in the NIR region beyond 650 nm.

**Fig. 3:**
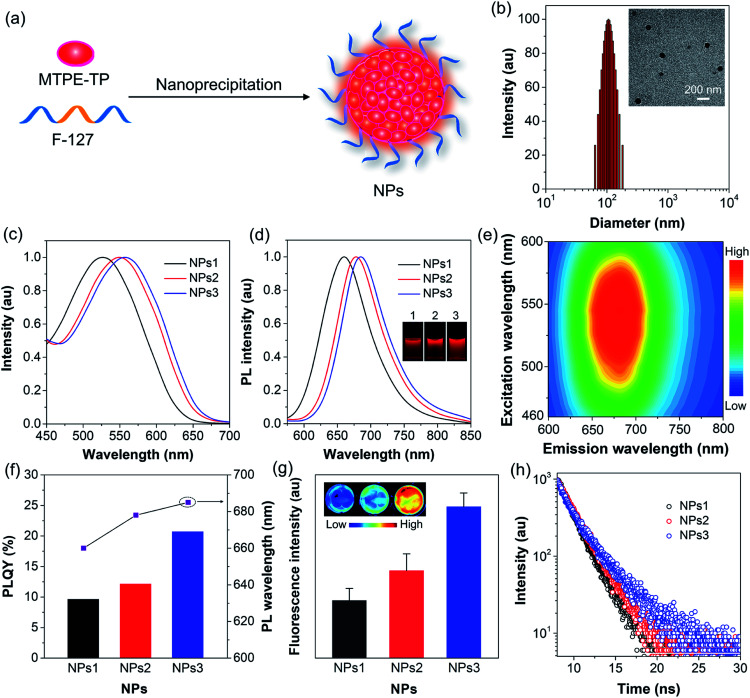
(a) Schematic illustration of nanoprecipitation to form NPs. (b) Representative DLS and TEM results of NPs3. (c) Absorption and (d) PL spectra of NPs1–3. The inset shows the photographs of NPs1–3 under UV light (365 nm) irradiation. (e) PLE map of NPs3 in aqueous dispersion. (f) PLQYs and maximal PL wavelengths of NPs1–3. (g) Fluorescence images and intensity of NPs1–3 (10^−5^ M) obtained from an IVIS system under 535 nm excitation. (h) Fluorescence decay curves of NPs1–3.

To endow the hydrophobic compounds with good water solubility and biocompatibility, the nanoprecipitation method (Fig. 3a) was adopted to formulate the self-assembled AIE NPs. With the assistance of an amphiphilic polymer surfactant, Pluronic F-127, uniform and stable organic NPs were obtained. The morphology and size of the NPs were characterized by dynamic light scattering (DLS) and transmission electron microscopy (TEM). DLS measurements reveal that the NPs formed by **MTPE-TP1–3** (NPs1–3) exhibit similar average diameters of about 110 nm (Fig. 3b, S27,† and Table 1), while TEM images suggest a kind of spherical morphology with an average diameter of about 90 nm. As compared to the solution state, both the absorption and emission spectra of NPs redshift (Fig. 3c and d). The PL maxima of NPs1–3 are 660 nm, 678 nm, and 685 nm, respectively, which are located in the NIR tissue transparent window and favourable for biological imaging. The large Stokes shift of about 130 nm suggests low self-absorption, a quality that is highly desirable for fluorescence imaging. PL excitation (PLE) mapping of NPs3 (Fig. 3e) indicates that it can be excited efficiently by the available excitation light (*e.g.*, 535 nm) of an *in vivo* imaging system (IVIS). The photographs of the NP solutions under 365 nm UV light irradiation reveal very bright emission. As shown in Fig. 3f and Table 1, the PLQYs of NPs1–3 are measured to be 9.6%, 12.1%, and 20.7%, respectively. The comparison of some organic/polymer NIR NPs with similar emission wavelengths is displayed in Table S1,† suggesting high brightness of NPs3. The PL intensity obtained from the IVIS and corresponding images (Fig. 3g) also manifests that the fluorescence brightness follows the order: NPs3 > NPs2 > NPs1. It is worth noting that from **MTPE-TP1** to **MTPE-TP3**, the PL maximum red shifts for 25 nm, and the PLQY exhibits a more than two-fold increase. This phenomenon is different from most previous results which show that the non-radiative decay usually increases when the bandgap is reduced. In this work, the introduction of AIE blocks into the TP unit and therefore the hindrance of molecular motions in the aggregate form is considered to be the main reason for the high brightness.

**Table 1:** Summary of the photophysical properties of **MTPE-TP1–3**

Compound	*S* a (mg mL^−1^)	*λ*_abs_ b (nm)	*λ*_PL_ b (nm)	*Φ*_F_ b (%)	*λ*_PL_ c (nm)	*d*_DLS_ d (nm)	*λ*_abs_ d (nm)	*λ*_PL_ d (nm)	*Φ*_F_ d (%)	*τ* d ^,^ e (ns)	*k*_r_/*k*_nr_^d^ (×10^8^ s^−1^)
**MTPE-TP1**	0.58	518	658	7.9	662	113	526	660	9.6	1.96	0.49/4.61
**MTPE-TP2**	0.36	538	670	6.6	683	111	549	678	12.1	2.26	0.54/3.88
**MTPE-TP3**	1.22	543	673	7.8	694	106	555	685	20.7	3.25	0.64/2.43

a Solubility in DMSO.

b Measured in DMSO (10^−5^ M).

c Measured in solid powder.

d Measured in NPs.

e The detailed fluorescence lifetime fitting is presented in Table S2.

In order to uncover the photophysical processes of the simultaneously red-shifted emission and enhanced brightness, we measured the fluorescence lifetime of encapsulated NPs. As depicted in Fig. 3h, the lifetime increases from NPs1 to NPs3, which is in the same trend as PLQY. The fluorescence properties are closely linked to radiative and non-radiative decay rates (*k*_r_ and *k*_nr_) from the excited state to ground state. Their relationships can be expressed as *k*_r_ = *Φ*_F_/*τ*, and *k*_nr_ = 1/*τ* − *k*_r_, where *Φ*_F_ is the PLQY and *τ* is the fluorescence lifetime. Accordingly, the radiative/non-radiative decay rate constants of NPs1–3 are calculated to be 0.49/4.61 × 10^8^ s^−1^, 0.54/3.88 × 10^8^ s^−1^, and 0.64/2.43 × 10^8^ s^−1^, respectively (Table 1). Interestingly, the radiative decay rate increases slightly, while the non-radiative decay rate decreases a lot. The twisted molecular structure is beneficial for enhanced radiative decay, and more importantly, significantly inhibits the non-radiative process.^52,53^ This result clearly demonstrates that the non-radiative deactivation pathway is greatly suppressed by the restriction of molecular motions, which is effective for realizing highly bright luminogens.^54^

**Fig. 4:**
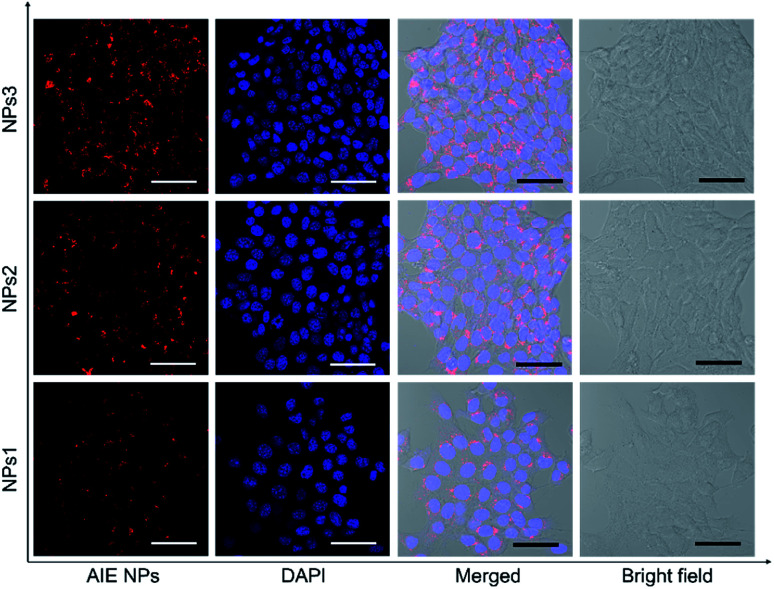
Confocal laser scanning microscope (CLSM) images of 4T1 breast cancer cells after incubation with NPs1–3 for 4 h. The cell nuclei were stained with DAPI (blue). Scale bars indicate 50 μm.

After studying the photophysical properties, we next exploited the application of the water-soluble NIR NPs in biological imaging both *in vitro* and *in vivo*. First, their potential in cellular imaging was studied with confocal laser scanning microscopy (CLSM). 4T1 breast cancer cells were respectively incubated with NPs1–3 in the same concentration (20 μM) for 4 h, and then the cells were fixed and the cell nuclei were stained with 4′,6-diamidino-2-phenylindole (DAPI). As depicted in Fig. 4, an intense red signal is obviously observed in the cell cytoplasm, demonstrating successful uptake of the NPs. It is noted that relatively weak red fluorescence is observed for the NPs1-treated cells, whereas the cells incubated with NPs3 at the same concentration exhibit robust red fluorescence signals. This result is in line with the PL brightness of the NPs. Moreover, little side effect in cell viability is observed as more than 90% of cells remain alive after treating with a high concentration (50 μM) of NPs (Fig. S28†). These results demonstrate that the bright NIR AIEgens, especially NPs3, offer great promise for fluorescence cell imaging.

**Fig. 5:**
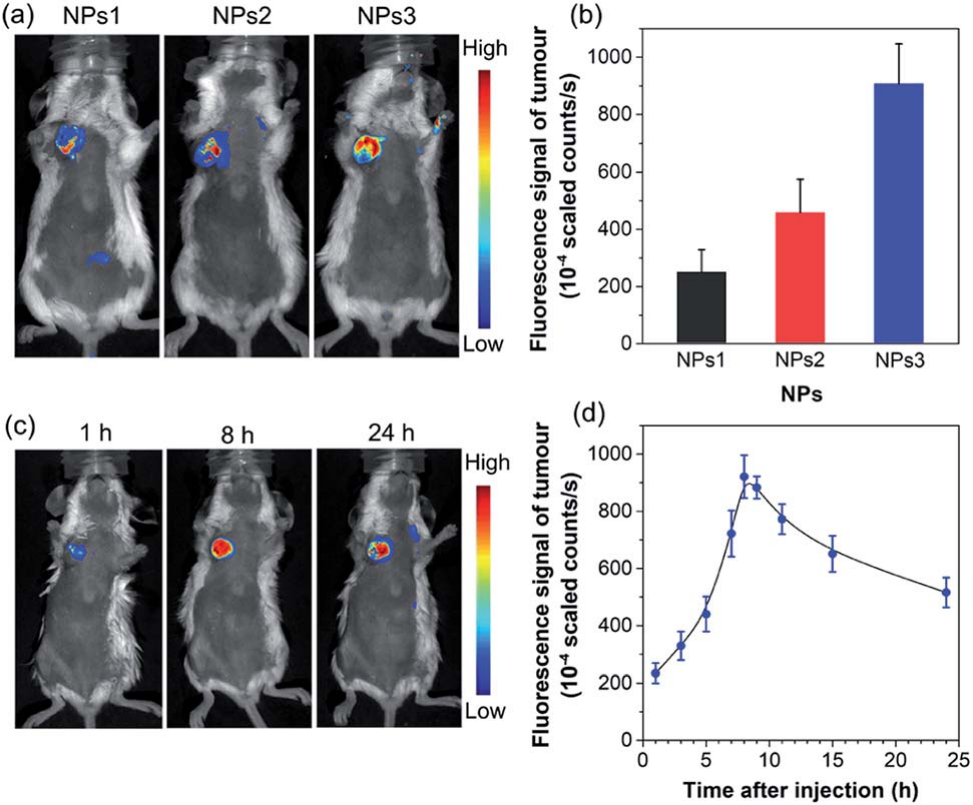
(a) *In vivo* non-invasive fluorescence imaging of tumour-bearing mice and (b) corresponding fluorescence intensity of the tumour site after intravenous injection of NPs1–3 for 8 h (*n* = 3). (c) *In vivo* fluorescence imaging of tumour-bearing mice and (d) corresponding fluorescence intensity of the tumour site at different time intervals after administrating NPs3 (*n* = 3).

To further assess the *in vivo* imaging performance of AIE NPs, non-invasive whole-body fluorescence imaging was conducted on 4T1 tumour-bearing mice. In this study, the same dose of NPs1–3 (200 μL, 500 μM based on AIEgens) was intravenously injected into tumour-bearing mice through the tail vein, respectively. As displayed in Fig. 5a, the tumour tissues significantly light up by the NPs, which clearly delineates the tumour from the surrounding normal tissues. In particular, the NPs3-treated mice exhibit much stronger tumour fluorescence than that of NPs1 and NPs2, which has also been confirmed by the *ex vivo* imaging results of resected tumours (Fig. S29†). The quantitative analysis (Fig. 5b) verifies that the fluorescence intensity of NPs3 in the tumour site is about 1.9-fold higher than that of NPs2, and 3.6 times greater than that of NPs1, which is consistent with the *in vitro* cellular imaging results and PLQY data. The best tumour imaging performance of NPs3 is mainly ascribed to the longer emission wavelength and higher PLQY. This result manifests that **MTPE-TP3** can be used as a highly efficient NIR probe for realizing high-contrast biomedical imaging *in vivo*.

After verifying the greater potency of NPs3 for bioimaging than the other two NPs, we next explored the detailed time-dependent tumour accumulation and imaging performance *in vivo*. The fluorescence images of mice were recorded at different time points after the administration of NPs3. As shown in the representative images in Fig. 5c, and the corresponding fluorescence intensity of the tumour site in Fig. 5d, the NPs exhibit a tendency to accumulate into the tumour with time elapsing, suggesting a good tumour preferential profile. The maximal tumour signal is observed at about 8 h post tail vein injection, which then gradually decreases. However, even after 24 hours, the tumour site is still highly visualized, and the strong fluorescent signal makes the tumour clearly distinguishable from normal tissues. The efficient accumulation of AIE NPs at the tumour site is mainly due to the enhanced permeability and retention (EPR) effect.^55–57^ The results show that the bright NIR NPs obtained through rational molecular design hold great promise for precise non-invasive tumour diagnosis in a high contrast manner.

## Conclusions

In summary, we demonstrate for the first time that AIE building blocks (*e.g.*, TPE) could efficiently extend the conjugation, boost the brightness, and enhance the solubility of organic NIR fluorophores at the same time. After introducing two TPE groups, the absorption/emission wavelength red shifts with the extension of the conjugation length, the brightness increases greatly because of the suppressed non-radiative processes, and the solubility also increases since the molecular rotors disrupt the intermolecular interaction, suggesting that the AIE unit works better than the typical solubility enhancer, bulky alkyl chains. Both *in vitro* cellular imaging and *in vivo* tumour imaging results verify that NIR AIE NPs are efficient for biomedical imaging, and the reddest and brightest **MTPE-TP3** NPs give the best tumour diagnostic outcome. This work highlights that AIEgens could serve as a potent regulator to obtain an optimal emission wavelength, brightness, and solubility of organic emitters, being useful for designing molecular probes.

## Experimental section

### General methods

All the chemicals and reagents were purchased from chemical sources and were used as received. Nuclear magnetic resonance (NMR) spectra were recorded on a Bruker AV 400 spectrometer. High-resolution mass spectra (HRMS) were measured with a GCT premier CAB048 mass spectrometer in matrix assisted laser desorption ionization-time of flight (MALDI-TOF) mode. The geometry optimization was performed at the level of B3LYP/6-31G* using the density functional theory (DFT) method with the Gaussian 09 program package (Cartesian coordinates see Tables S3–S5†). The UV-vis absorption spectra were recorded using a Shimadzu 2550 UV-vis scanning spectrophotometer. Steady-state photoluminescence (PL) spectra were recorded on a Horiba Fluorolog-3 spectrofluorometer. The PLQY was measured using a Hamamatsu absolute PL quantum yield spectrometer C11347 Quantaurus-QY. Transient PL at room temperature was measured using an Edinburgh FLSP980 fluorescence spectrophotometer. Dynamic light scattering (DLS) was measured on a 90 plus particle size analyser. Transmission electron microscope (TEM) images were acquired from a JEM-2010F transmission electron microscope with an accelerating voltage of 200 kV.

### Synthesis of **MTPE-TP1**

2,5-Bis(4-(2,2-bis(4-methoxyphenyl)-1-phenylvinyl)phenyl)thiophene-3,4-diamine (0.54 g, 0.6 mmol) and 1,4-dioxane-2,3-diol (0.12 g, 1 mmol) were dissolved in a mixture of chloroform (20 mL) and acetic acid (20 mL) in a 100 mL flask. The reaction mixture was heated to 60 °C, and stirred for 12 h. Then water was added, and the mixture was extracted with dichloromethane three times. The organic phase was combined and dried with anhydrous MgSO_4_. After the removal of the solvent under reduced pressure, the residue was purified by column chromatography on silica gel using dichloromethane/hexane (v/v 1 : 2) as the eluent to afford 5,7-bis(4-(2,2-bis(4-methoxyphenyl)-1-phenylvinyl)phenyl)thieno[3,4-*b*]pyrazine (**MTPE-TP1**) as a red solid (76% yield). ^1^H NMR (400 MHz, CDCl_3_, 25 °C) *δ* (ppm): 8.46 (s, 2H), 7.93 (d, 4H), 7.13–7.04 (m, 14H), 7.02 (d, 4H), 6.95 (d, 4H), 6.69–6.62 (m, 8H), 3.74 (d, 12H). ^13^C NMR (100 MHz, CDCl_3_, 25 °C) *δ* (ppm): 158.28, 158.14, 144.19, 144.13, 143.81, 140.66, 140.16, 138.69, 136.35, 136.27, 132.67, 132.65, 132.00, 131.97, 131.53, 130.76, 127.77, 127.02, 126.21, 113.23, 113.00, 55.11, 55.10. HRMS (MALDI-TOF) *m*/*z*: [M]^+^ calcd for C_62_H_48_N_2_O_4_S, 916.3335; found, 916.3342.

### Synthesis of **MTPE-TP2**

2,5-Bis(4-(2,2-bis(4-methoxyphenyl)-1-phenylvinyl)phenyl)thiophene-3,4-diamine (0.54 g, 0.6 mmol) and benzil (0.21 g, 1 mmol) were dissolved in a mixture of chloroform (20 mL) and acetic acid (20 mL) in a 100 mL flask. The reaction mixture was heated to 60 °C, and stirred for 12 h. Then water was added, and the mixture was extracted with dichloromethane three times. The organic phase was combined, and dried with anhydrous MgSO_4_. After the removal of the solvent under reduced pressure, the residue was purified by column chromatography on silica gel using dichloromethane/hexane (v/v 1 : 2) as the eluent to afford 5,7-bis(4-(2,2-bis(4-methoxyphenyl)-1-phenylvinyl)phenyl)-2,3-diphenylthieno[3,4-*b*]pyrazine (**MTPE-TP2**) as a dark red solid (72% yield). ^1^H NMR (400 MHz, CDCl_3_, 25 °C) *δ* (ppm): 8.07 (d, 4H), 7.50 (d, 4H), 7.37–7.27 (m, 6H), 7.15–7.05 (m, 14H), 7.02 (d, 4H), 6.95 (d, 4H), 6.70–6.61 (m, 8H), 3.73 (d, 12H). ^13^C NMR (100 MHz, CDCl_3_, 25 °C) *δ* (ppm): 158.29, 158.12, 152.17, 144.23, 143.91, 140.57, 139.30, 138.79, 136.42, 136.31, 132.70, 132.68, 131.95, 131.61, 131.19, 131.05, 129.87, 128.84, 128.07, 127.77, 126.81, 126.20, 113.22, 113.00, 55.11, 55.10. HRMS (MALDI-TOF) *m*/*z*: [M]^+^ calcd for C_74_H_56_N_2_O_4_S, 1068.3961; found, 1068.3969.

### Synthesis of **MTPE-TP3**

5,7-Bis(4-(2,2-bis(4-methoxyphenyl)-1-phenylvinyl)phenyl)-2,3-bis(4-bromophenyl)thieno[3,4-*b*]pyrazine (0.49 g, 0.4 mmol), 4,4,5,5-tetramethyl-2-(1,2,2-triphenylvinyl)-1,3,2-dioxaborolane (0.38 g, 1 mmol), and Pd(PPh_3_)_4_ (35 mg, 0.03 mmol) were added into a 100 mL Schlenk flask. The flask was then vacuumed and purged with nitrogen three times, and THF (30 mL) and aqueous K_2_CO_3_ solution (10 mL, 2.0 M) were added. Then the mixture was heated to reflux, and stirred for 24 h. After cooling to room temperature, water was added, and the resulting mixture was extracted with dichloromethane three times. The organic phase was combined, and dried with anhydrous MgSO_4_. After the removal of the solvent under reduced pressure, the residue was purified by column chromatography on silica gel using dichloromethane/hexane (v/v 1 : 2) as the eluent to result in 5,7-bis(4-(2,2-bis(4-methoxyphenyl)-1-phenylvinyl)phenyl)-2,3-bis(4-(1,2,2-triphenylvinyl)phenyl)thieno[3,4-*b*]pyrazine (**MTPE-TP3**) as a dark red solid (73% yield). ^1^H NMR (400 MHz, CDCl_3_, 25 °C) *δ* (ppm): 8.00 (s, 2H), 7.25–6.79 (m, 62H), 6.72–6.64 (m, 8H), 3.73 (d, 12H). HRMS (MALDI-TOF) *m*/*z*: [M]^+^ calcd for C_114_H_84_N_2_O_4_S, 1577.6185; found, 1577.6093.

### Preparation of the NPs

1 mM of AIEgens and 4 mg of Pluronic F-127 were dissolved in 1 mL of tetrahydrofuran (THF). The obtained THF solution was poured into 10 mL of deionized water under sonication with a microtip probe sonicator (XL2000, Misonix Incorporated, NY). Subsequently, the mixture was sonicated for another 1 min and violently stirred in a fume hood overnight at room temperature to evaporate residue THF, and the NP solution was used directly. The concentration of the NPs was estimated based on the initial feeding AIEgen in the nanoprecipitation process, *i.e.*, the concentration of AIEgens in the solution.

### Cell culture

4T1 breast cancer cells were cultured in Dulbecco's Modified Eagle's Medium (DMEM) containing 10% fetal bovine serum (FBS) and 1% penicillin–streptomycin at 37 °C in a humidified environment containing 5% CO_2_. Before experiments, the cells were precultured until confluence was reached.

### Cellular imaging

4T1 breast cancer cells were seeded and grown on a 35 mm Petri dish with a cover slip at a density of about 2 × 10^5^ cells per well in 2 mL of culture medium. The cells were incubated respectively with NPs1–3 (final concentration: 20 μM based on AIEgens) in the medium for 4 h. Then the cells were washed with 1× PBS three times, fixed in 4% paraformaldehyde for 20 min, washed three times with 1× PBS, and incubated with 4′,6-diamidino-2-phenylindole (DAPI) for 10 min. The cells were then washed three times with 1× PBS. Fresh PBS was added to the confocal chambers, and laser scanning confocal microscopy (Zeiss LSM 710, Jena, Germany) was performed (*λ*_ex_ = 405 nm for DAPI, *λ*_ex_ = 543 nm for NPs1–3; fluorescent signals were collected at 630–760 nm for NPs1–3 and 430–475 nm for DAPI, respectively).

### Cytotoxicity study

3-(4,5-Dimethyl-2-thiazolyl)-2,5-diphenyl-tetrazolium bromide (MTT) assay was used to evaluate the cytotoxicity of the NPs. 4T1 breast cancer cells were harvested in a logarithmic growth phase and seeded in 96-well plates at a density of about 4 × 10^4^ cells per well for 24 h. Then different concentrations (0, 2.5, 5, 10, 20, and 50 μM based on AIEgens) of NPs1–3 were added into the cell culture medium separately. After incubating for 24 h, the culture medium was replaced with a fresh medium containing 0.5 mg mL^−1^ of MTT, and incubated for 4 h. The culture medium was discarded and replaced with 100 μL of DMSO with gentle shaking. Afterwards, the absorbance of MTT at 490 nm was measured using a microplate reader (GENios Tecan). Cell viability was expressed by the ratio of the absorbance of cells incubated with NPs to that of the cells incubated with the culture medium only.

### Animal experiments

All animal studies were conducted under the guidelines set by the Tianjin Committee of Use and Care of Laboratory Animals, and the overall project protocols were approved by the Animal Ethics Committee of Nankai University.

### Tumour-bearing mice

6 week-old female BALB/c mice were obtained from the Laboratory Animal Centre of the Academy of Military Medical Sciences (Beijing, China). To establish the xenograft 4T1 tumour-bearing mouse model, 4T1 breast cancer cells (1 × 10^6^) suspended in 30 μL of RPMI-1640 medium were injected subcutaneously into the right axillary space of the BALB/c mouse. After 9 days, the mice with tumour volumes of about 80–120 mm^3^ were used subsequently.

### *In vivo* fluorescence imaging

The xenograft 4T1 tumour-bearing mice were randomly selected for the following fluorescence imaging experiments. The tumour-bearing mice were anesthetized using 2% isoflurane in oxygen, and the NPs (200 μL, 500 μM based on AIEgens) were intravenously injected into the tumour-bearing mice using a microsyringe, respectively (*n*  = 3 mice for each group). *In vivo* fluorescence imaging was performed on a Maestro EX fluorescence imaging system (CRi, Inc.) with 535 nm excitation and signal collection in the spectral region of 600–850 nm.

## Author contributions

B. Z. T., D. D., and J. Q. conceived and designed the study. J. Q. synthesized and characterized the compounds. J. Q. and X. D. contributed equally to this work. J. Q. and X. D. performed the NP preparation and *in vitro* experiments. D. X., S. J., and C. C. performed the *in vivo* experiments. Y. C. performed the theoretical calculations. J. Q., X. D., Z. Z., Y. L., H.-Q. P., R. T. K. K., J. W. Y. L., D. D., and B. Z. T. analysed the data and participated in the discussion. J. Q., X. D., D. D., and B. Z. T. contributed to the writing of this paper. All authors contributed to the review and revision of this paper.

## Conflicts of interest

There are no conflicts to declare.

## Supplementary Material

SC-011-D0SC03423A-s001
