# Supplementary material for: Simultaneously boosting the conjugation, brightness and solubility of organic fluorophores by using AIEgens
Source: Chem Sci. 2020 Jul 27;11(32):8438–47. doi: 10.1039/d0sc03423a (PMC8163428; doi:10.1039/d0sc03423a)
Supplement: SC-011-D0SC03423A-s001 [file SC-011-D0SC03423A-s001.pdf]

Electronic Supplementary Material (ESI) for Chemical Science.
This journal is © The Royal Society of Chemistry 2020

Electronic Supplementary Information

Simultaneously boosting conjugation, brightness and solubility of organic fluorophore by AIEgen

Ji Qi, Xingchen Duan, Yuanjing Cai, Shaorui Jia, Chao Chen, Zheng Zhao, Ying Li, Hui-Qing Peng, Ryan T. K. Kwok, Jacky W. Y. Lam, Dan Ding and Ben Zhong Tang**

Syntheses and characterizations

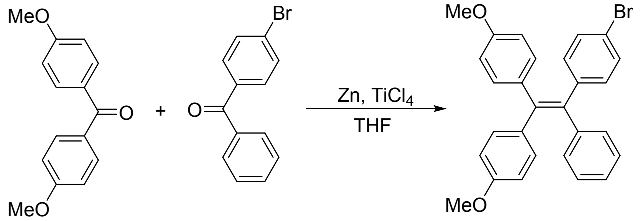

Synthesis of 4,4'-(2-(4-bromophenyl)-2-phenylethene-1,1-diyl)bis(methoxybenzene) (**3**)

Into a 500 mL of Schlenk flask, bis(4-methoxyphenyl)methanone (9.68 g, 40.0 mmol), (4-bromophenyl)(phenyl)methanone (10.44 g, 40.0 mmol) and zinc powder (10.4 g, 160 mmol) were added. The flask was then vacuumed and purged with dry nitrogen three times, and anhydrous THF (200 mL) was added. Then the mixture was cooled with ice-water to 0 °C, and $TiCl_4$ (13.2 mL, 120 mmol) was dropped into the mixture with rapid stirring. Then the mixture was heated to reflux, and stirred for 8 h. After cooling down to room temperature, the reaction was quenched by the adding aqueous HCl (1 M) and stirred for 2 h. And the mixture was extracted with dichloromethane for three times. The organic phase was combined, and dried with anhydrous $MgSO_4$. After removal of the solvent under reduced pressure, the residue was purified by column chromatography on silica gel using dichloromethane/hexane (v/v 1:3) as the eluent to result in 2-(4-(2,2-bis(4-methoxyphenyl)-1-phenylvinyl)phenyl)-4,4,5,5-tetramethyl-1,3,2-dioxaborolane as a white solid (38% yield). ^{1}H NMR (400 MHz, $CDCl_3$, 25 ºC) δ (ppm): 7.21 (d, 2H), 7.11 (d, 3H), 7.00 (d, 2H), 8.95-6.84 (m, 6H), 6.68-6.59 (m, 4H), 3.75 (d, 6H). ^{13}C NMR (400 MHz, $CDCl_3$, 25 ºC) δ (ppm): 158.31, 158.22, 143.81, 143.32, 140.81, 137.93, 136.04, 135.95, 133.05, 132.56, 132.53, 131.35, 130.87, 127.82, 127.68, 126.32, 120.03, 113.23, 113.05, 55.13, 55.10.

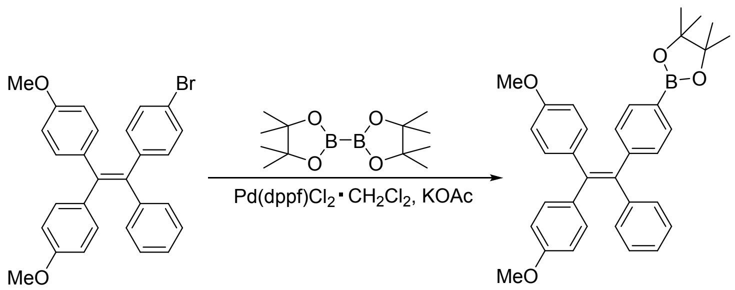

Synthesis of 2-(4-(2,2-bis(4-methoxyphenyl)-1-phenylvinyl)phenyl)-4,4,5,5-tetramethyl-1,3,2-dioxaborolane (**4**)

4,4'-(2-(4-Bromophenyl)-2-phenylethene-1,1-diyl)bis(methoxybenzene) (5.65 g, 12 mmol), bis(pinacolato)diboron (9.14 g, 36 mmol), Pd(dppf)Cl_2·CH_2Cl_2 (490 mg, 0.6 mmol), and KOAc (7.35 g, 75 mmol) were dissolved in 1,4-dioxane (80 mL) in a 250 mL of Schlenk flask. The flask was then vacuumed and purged with dry nitrogen three times. The reaction mixture was heated to 100 ^{o}C, and stirred for 24 h. Then water was added, and the mixture was extracted with ethyl acetate for three times. The organic phase was combined, and dried with anhydrous $MgSO_4$. After removal of the solvent under reduced pressure, the residue was purified by column chromatography on silica gel using dichloromethane/hexane (v/v 1:2) as the eluent to result in 2-(4-(2,2-bis(4-methoxyphenyl)-1-phenylvinyl)phenyl)-4,4,5,5-tetramethyl-1,3,2-dioxaborolane as a light yellow solid (87% yield). ^{1}H NMR (400 MHz, $CDCl_3$, 25 ºC) δ (ppm): 7.54 (d, 2H), 7.11-7.05 (m, 3H), 7.05-6.97 (m, 4H), 6.93 (m, 4H), 6.63 (d, 4H), 3.74 (d, 6H), 1.26 (s, 12H). ^{13}C NMR (100 MHz, $CDCl_3$, 25 ºC) δ (ppm): 158.16, 158.13, 147.42, 144.14, 140.60, 139.14, 136.31, 136.20, 134.11, 132.59, 131.41, 130.77, 127.67, 126.11, 113.09, 113.00, 83.66, 55.07, 55.05, 25.04.

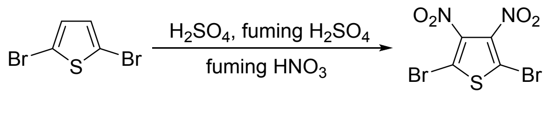

Synthesis of 2,5-dibromo-3,4-dinitrothiophene (**5**)

The mixture of 2,5-dibromothiophene (8.82 g, 50 mmol), sulfuric acid (75 mL), fuming sulfuric acid (25 mL), and fuming nitric acid (60 mL) was stirred at 0 ^oC for 5 h to finish the nitration reaction. Afterwards, the mixture was poured into the mixture of ice and water (600 mL) slowly to get a suspension, and filtered through a Buchner funnel, washed with water several times, and dried in vacuum to get 2,5-dibromo-3,4-dinitrothiophene as a white powder (90% yield). ^{13}C NMR (100 MHz, DMSO-d_6, 25 ºC) δ (ppm): 140.10, 117.15.

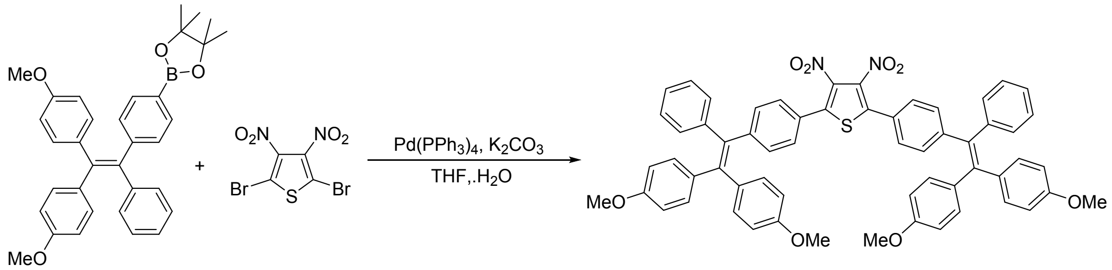

Synthesis of 2,5-bis(4-(2,2-bis(4-methoxyphenyl)-1-phenylvinyl)phenyl)-3,4-dinitrothiophene (**6**)

Into a 100 mL of Schlenk flask, 2-(4-(2,2-bis(4-methoxyphenyl)-1-phenylvinyl)phenyl)-4,4,5,5-tetramethyl-1,3,2-dioxaborolane (2.31 g, 10 mmol), 2,5-dibromo-3,4-dinitrothiophene (1.49 g, 4.5 mmol) and $Pd(PPh_3)_4$ (347 mg, 0.3 mmol) were added. The flask was then vacuumed and purged with nitrogen three times, and THF (30 mL) and aqueous K_2CO_3 solution (10 mL, 2.0 M) were added. Then the mixture was heated to reflux, and stirred for 24 h. After cooling to room temperature, water was added, and the resulting mixture was extracted with dichloromethane three times. The organic phase was combined, and dried with anhydrous $MgSO_4$. After the removal of the solvent under reduced pressure, the residue was purified by column chromatography on silica gel using dichloromethane/hexane (v/v 1:3) as the eluent to result in 2,5-bis(4-(2,2-bis(4-methoxyphenyl)-1-phenylvinyl)phenyl)-3,4-dinitrothiophene as an orange solid (83% yield). ^1H

NMR (400 MHz, $CDCl_3$, 25 ºC) δ (ppm): 7.21 (d, 4H), 7.18-7.06 (m, 10H), 7.04 (d, 4H), 6.97-6.88 (m, 8H), 6.71-6.59 (m, 8H), 3.75 (d, 12H). ^{13}C NMR (400 MHz, $CDCl_3$, 25 ºC) δ (ppm): 158.59, 158.38, 147.33, 143.53, 142.02, 140.39, 137.75, 136.57, 135.76, 135.61, 132.67, 132.59, 132.08, 131.41, 128.22, 127.96, 126.49, 125.51, 113.29, 113.08, 55.18, 55.11.

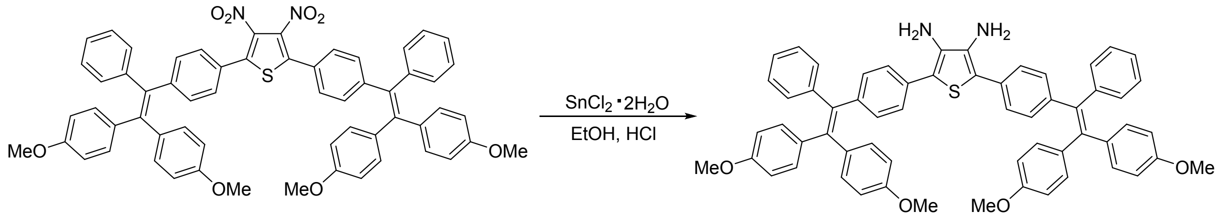

Synthesis of 2,5-bis(4-(2,2-bis(4-methoxyphenyl)-1-phenylvinyl)phenyl)thiophene-3,4-diamine (**7**)

2,5-Bis(4-(2,2-bis(4-methoxyphenyl)-1-phenylvinyl)phenyl)-3,4-dinitrothiophene (2.87 g, 3 mmol) was suspended in a solution of 60 mL of ethanol and 40 mL of concentrated HCl in a 250 mL flask. Under vigorous stirring, 18 g of $SnCl_2·2H_2O$ was added in three portions. Then the reaction mixture was heated to reflux, and stirred for 20 h. After cooling down to room temperature, the reaction mixture was poured into 25 wt% KOH aqueous solution. Dichloromethane was used to extract the product. The organic phase was separated, combined and dried over anhydrous sodium sulfate. The solvent was removed under reduced pressure to afford 2,5-bis(4-(2,2-bis(4-methoxyphenyl)-1-phenylvinyl)phenyl)thiophene-3,4-diamine as a dark red condensed liquid, and used without further purification. ^1H NMR (400 MHz, $CDCl_3$, 25 ºC) δ (ppm): 7.22 (d, 4H), 7.16-7.08 (m, 6H), 7.08-7.01 (m, 8H), 7.00-6.90 (m, 8H), 6.69-6.60 (m, 8H), 3.74 (m, 12H). ^{13}C NMR (400 MHz, $CDCl_3$, 25 ºC) δ (ppm): 158.20, 158.10, 144.18, 142.68, 140.42, 138.69, 136.35, 136.31, 132.64, 132.61, 132.13, 131.88, 131.48, 127.77, 126.50, 126.20, 117.08, 113.18, 113.02, 55.13, 55.10.

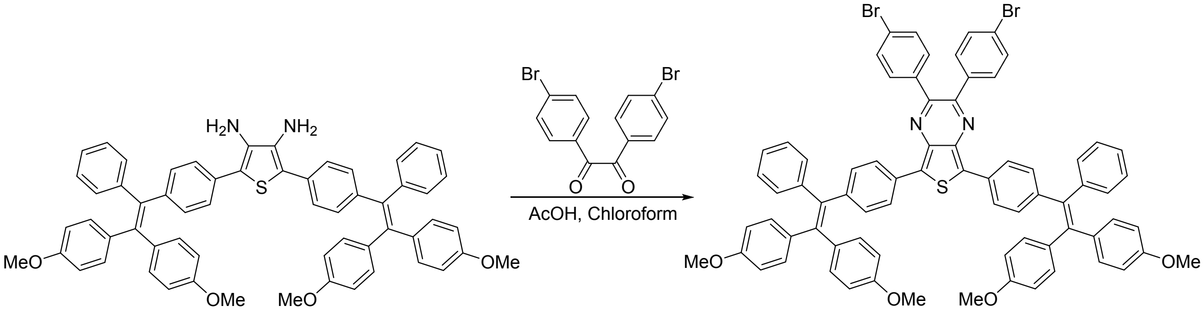

Synthesis of 5,7-bis(4-(2,2-bis(4-methoxyphenyl)-1-phenylvinyl)phenyl)-2,3-bis(4-bromophenyl)thieno[3,4-*b*]pyrazine (**12**)

2,5-Bis(4-(2,2-bis(4-methoxyphenyl)-1-phenylvinyl)phenyl)thiophene-3,4-diamine (0.54 g, 0.6 mmol) and (0.37 g, 1 mmol) were dissolved in the mixture of chloroform (20 mL) and acetic acid (20 mL) in a 100 mL flask. The reaction mixture was heated to 60 ^{o}C, and stirred for 12 h. Then water was added, and the mixture was extracted with dichloromethane three times. The organic phase was combined, and dried with anhydrous $MgSO_4$. After the removal of the solvent under reduced pressure, the residue was purified by column chromatography on silica gel using dichloromethane/hexane (v/v 1:2) as the eluent to afford 5,7-bis(4-(2,2-bis(4-methoxyphenyl)-1-phenylvinyl)phenyl)-2,3-bis(4-bromophenyl)thieno[3,4-*b*]pyrazine as a red solid (75% yield). ^{1}H NMR (400 MHz, $CDCl_3$, 25 ºC) δ (ppm): 8.01 (d, 4H), 7.46 (d, 4H), 7.36 (d, 4H), 7.16-7.06 (m, 14H), 7.02 (d, 4H), 6.95 (d, 4H), 6.70-6.61 (m, 8H), 3.74 (d, 12H). ^{13}C NMR (100 MHz, $CDCl_3$, 25 ºC) δ (ppm): 158.32, 158.16, 150.59, 144.20, 144.15, 140.73, 138.69, 138.59, 137.91, 136.35, 132.72, 132.68, 132.53, 132.01, 131.60, 131.52, 131.46, 131.39, 131.31, 130.91, 127.83, 126.83, 126.26, 123.69, 113.24, 113.03, 55.12. HRMS (MALDI-TOF) *m*/*z*: $[M]^+$ calcd for $C_{74}H_{54}N_2O_4SBr_2$, 1226.2151; found, 1226.2123.

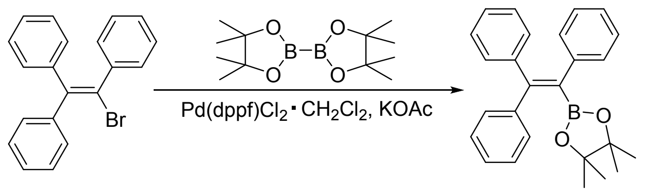

4,4,5,5-Tetramethyl-2-(1,2,2-triphenylvinyl)-1,3,2-dioxaborolane (**13**)

(2-Bromoethene-1,1,2-triyl)tribenzene (3.35 g, 10 mmol), bis(pinacolato)diboron (3.81 g, 15 mmol), Pd(dppf)Cl_2·CH_2Cl_2 (408 mg, 0.5 mmol) and KOAc (2.95 g, 30 mmol) were added into a 250 mL Schlenk flask. The flask was then vacuumed and purged with dry nitrogen three times, and 1,4-dioxane (60 mL) was added. The reaction mixture was heated to 100 °C, and stirred for 24 h. Then water was added, and the mixture was extracted with ethyl acetate for three times. The organic phase was combined, and dried with anhydrous $MgSO_4$. After the removal of the solvent under reduced pressure, the residue was purified by column chromatography on silica gel using dichloromethane/hexane (v/v 1:1) as the eluent to result in 4,4,5,5-tetramethyl-2-(1,2,2-triphenylvinyl)-1,3,2-dioxaborolane as a white solid (85% yield). ^{1}H NMR (400 MHz, $CDCl_3$, 25 °C) δ (ppm): 7.36-7.31 (m, 2H), 7.31-7.27 (m, 3H), 7.16-7.02 (m, 8H), 6.99-6.92 (m, 2H), 1.12 (s, 12H). ^{13}C NMR (100 MHz, $CDCl_3$, 25 °C) δ (ppm): 151.43, 144.68, 141.86, 141.72, 130.94, 129.74, 129.44, 129.17, 128.98, 128.75, 128.64, 128.01, 127.96, 127.59, 127.54, 127.17, 126.79, 125.86, 83.70, 24.56.

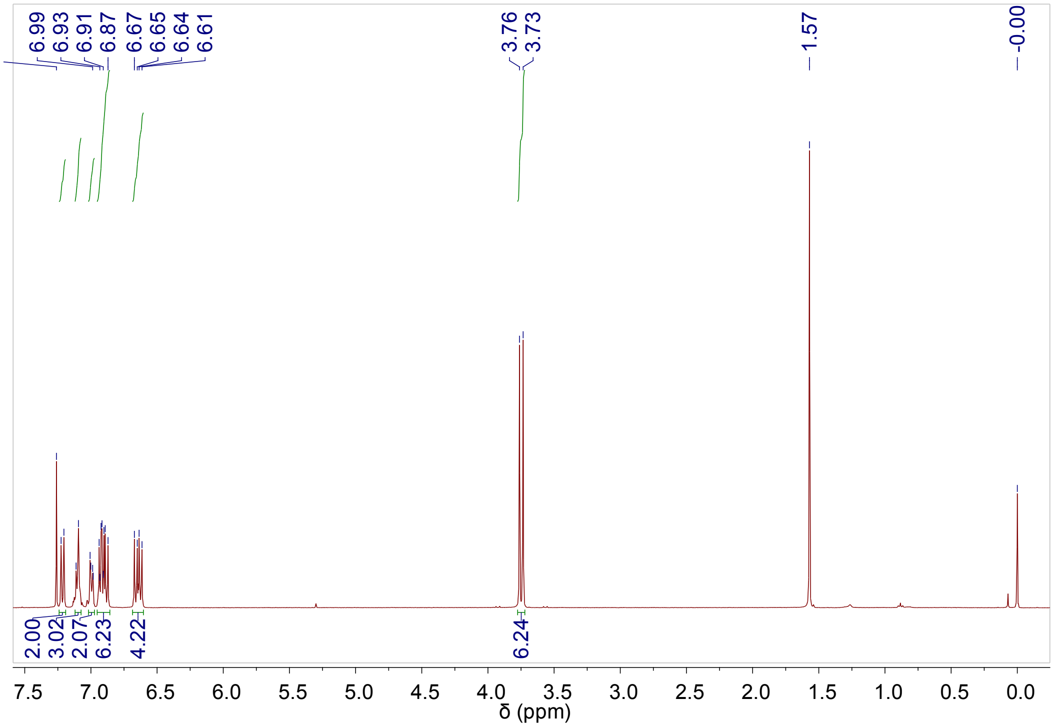

Fig. S1 ^{1}H NMR spectrum of 4,4'-(2-(4-bromophenyl)-2-phenylethene-1,1-diyl)bis(methoxybenzene) in $CDCl_3$ at 298 K.

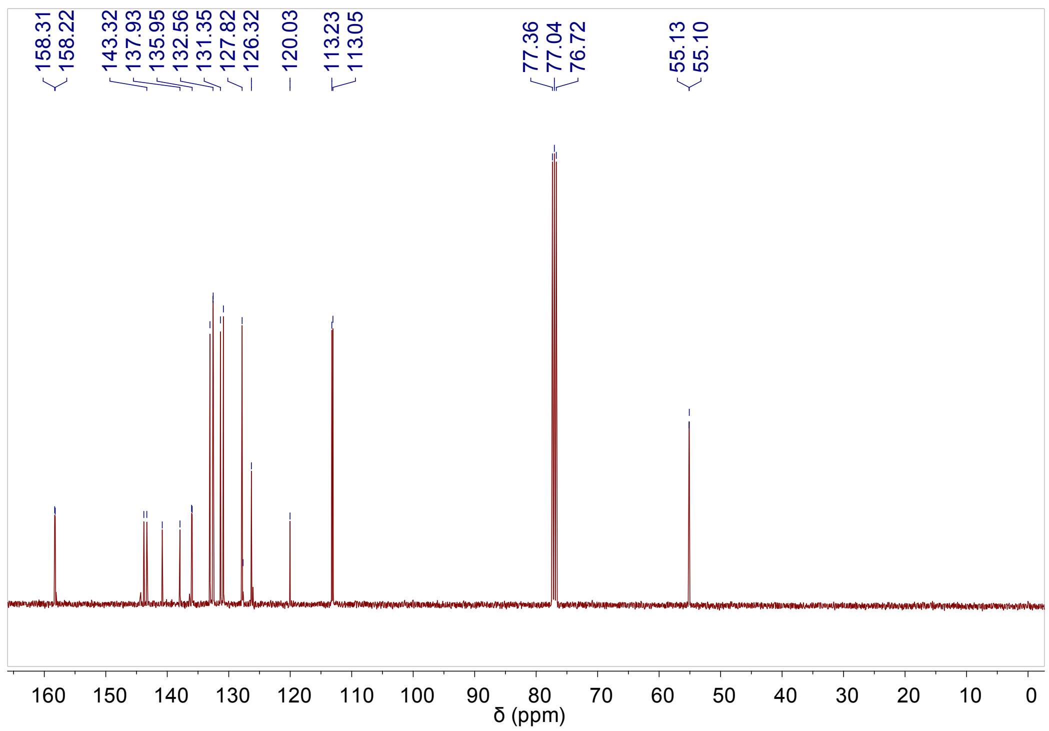

Fig. S2 ^{13}C NMR spectrum of 4,4'-(2-(4-bromophenyl)-2-phenylethene-1,1-diyl)bis(methoxybenzene) in $CDCl_3$ at 298 K.

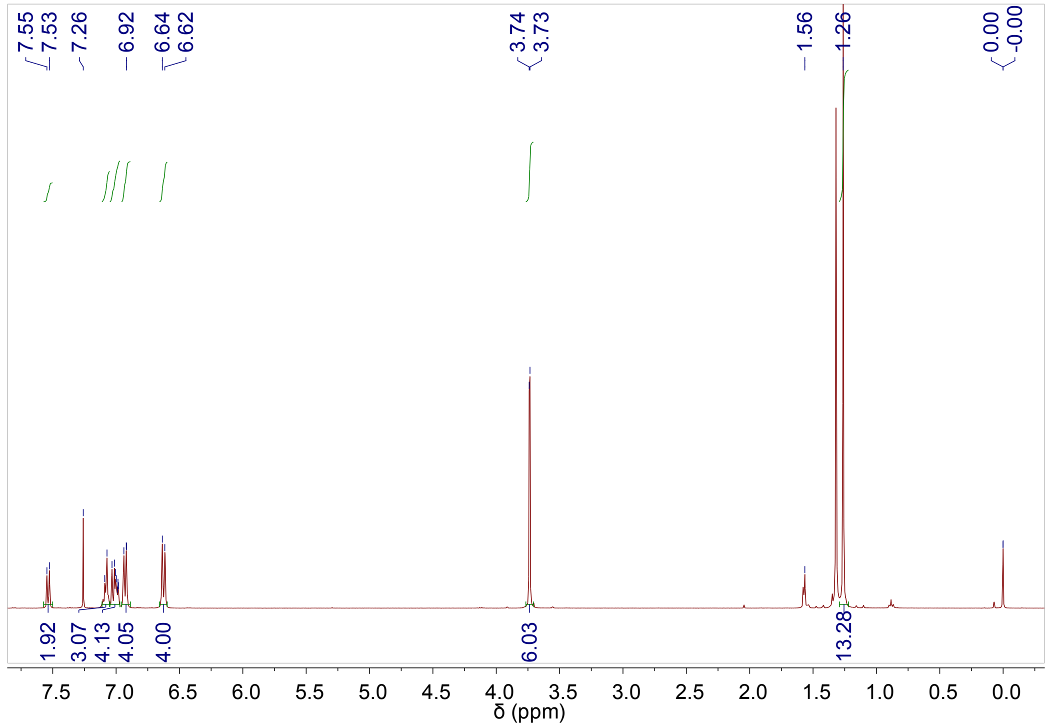

Fig. S3 ^{1}H NMR spectrum of 2-(4-(2,2-bis(4-methoxyphenyl)-1-phenylvinyl)phenyl)-4,4,5,5-tetramethyl-1,3,2-dioxaborolane in $CDCl_3$ at 298 K.

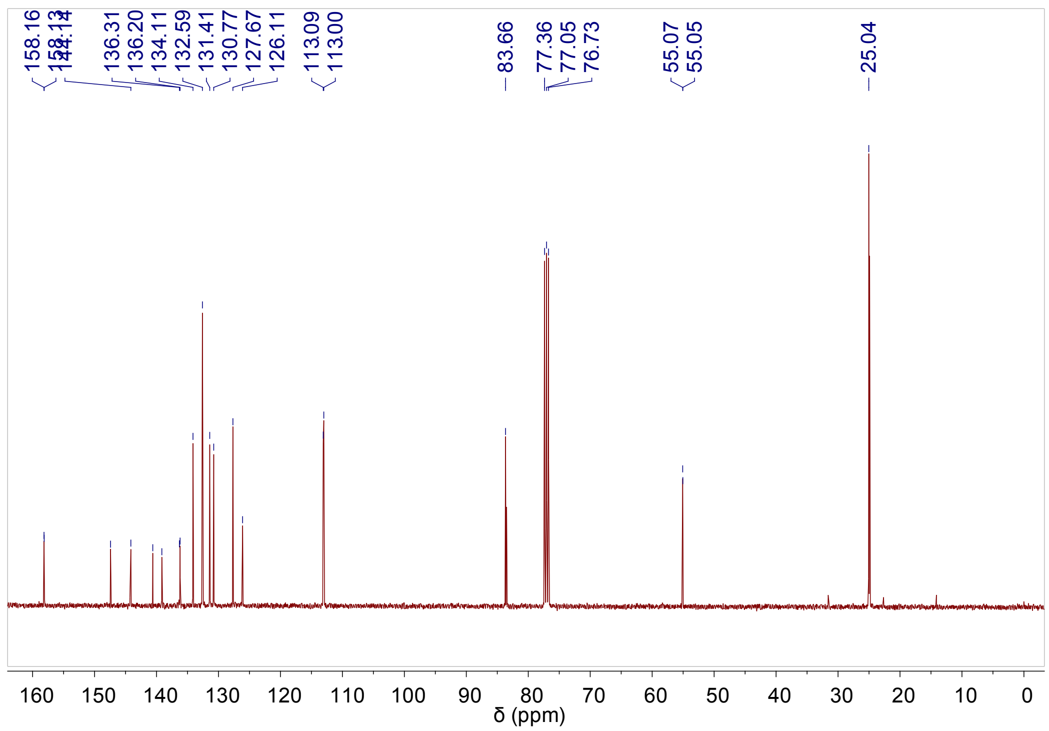

Fig. S4 ^{13}C NMR spectrum of 2-(4-(2,2-bis(4-methoxyphenyl)-1-phenylvinyl)phenyl)-4,4,5,5-tetramethyl-1,3,2-dioxaborolane in $CDCl_3$ at 298 K.

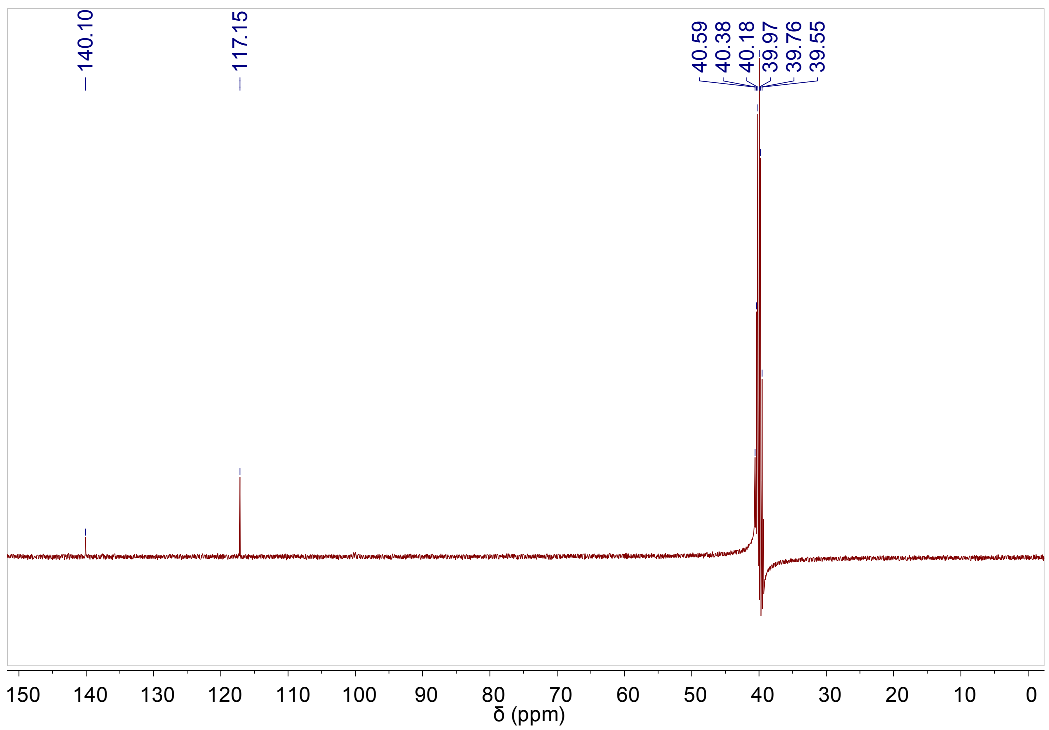

Fig. S5 ^{13}C NMR spectrum of 2,5-dibromo-3,4-dinitrothiophene in DMSO-d_6 at 298 K.

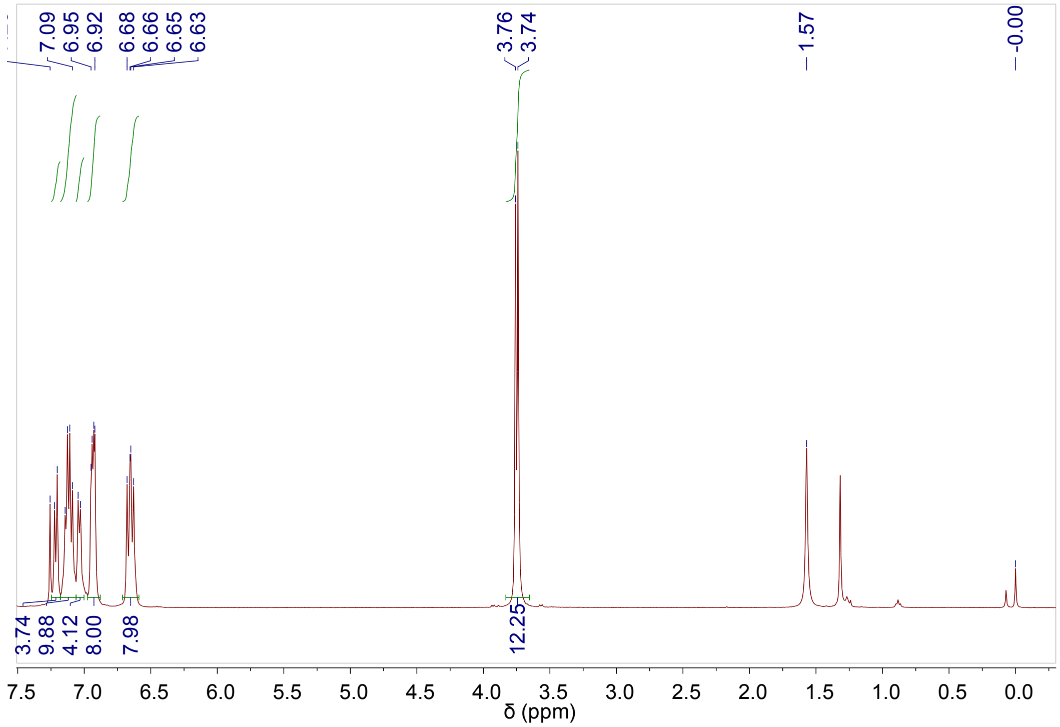

Fig. S6 ^{1}H NMR spectrum of 2,5-bis(4-(2,2-bis(4-methoxyphenyl)-1-phenylvinyl)phenyl)-3,4-dinitrothiophene in $CDCl_3$ at 298 K.

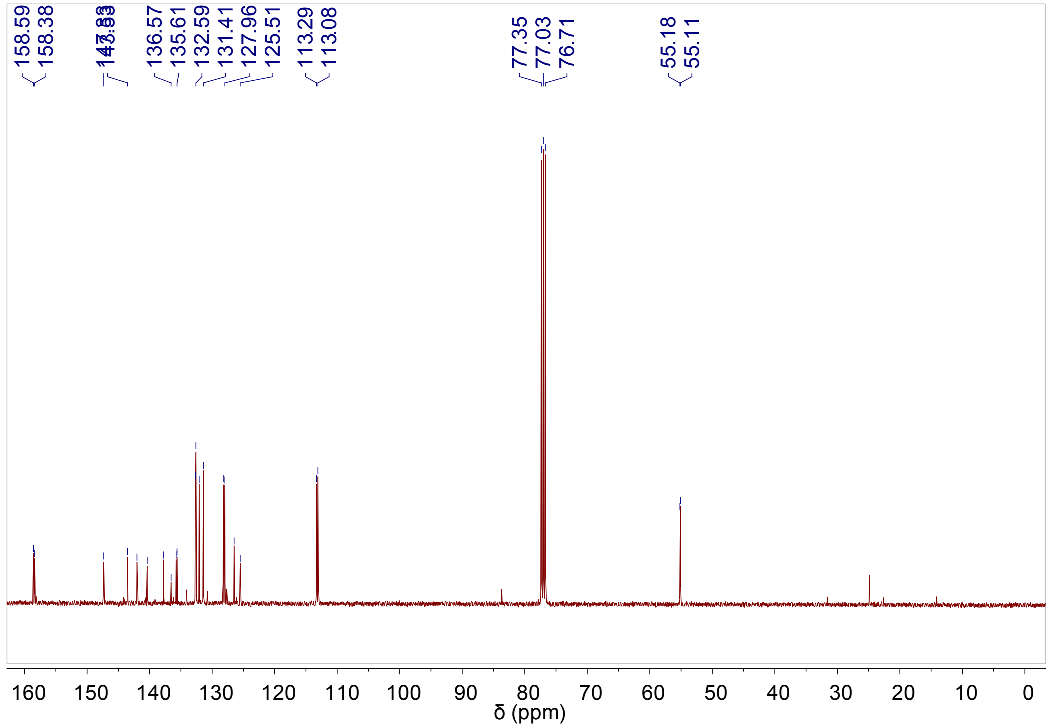

Fig. S7 ^{13}C NMR spectrum of 2,5-bis(4-(2,2-bis(4-methoxyphenyl)-1-phenylvinyl)phenyl)-3,4-dinitrothiophene in $CDCl_3$ at 298 K.

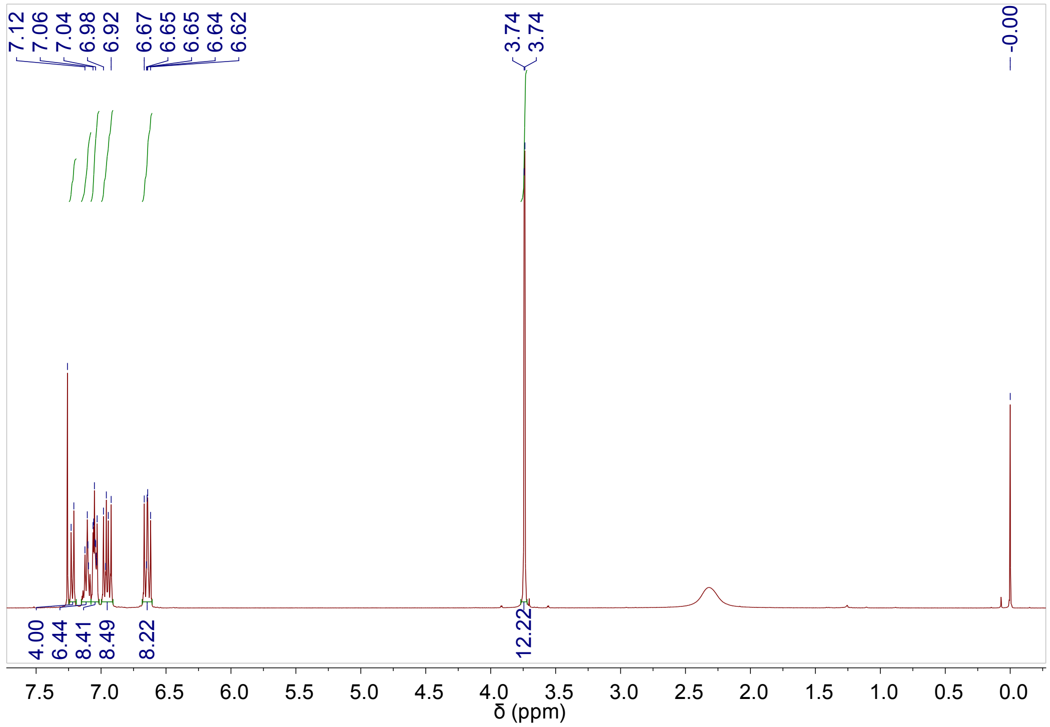

Fig. S8 ^{1}H NMR spectrum of 2,5-bis(4-(2,2-bis(4-methoxyphenyl)-1-phenylvinyl)phenyl)thiophene-3,4-diamine in $CDCl_3$ at 298 K.

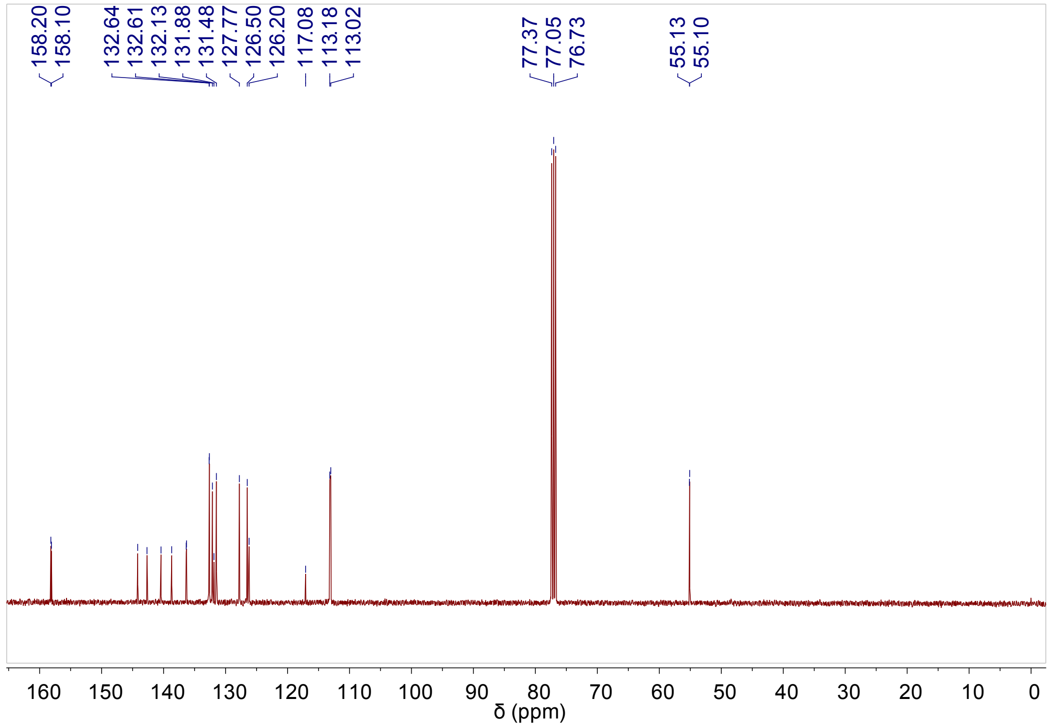

Fig. S9 ^{13}C NMR spectrum of 2,5-bis(4-(2,2-bis(4-methoxyphenyl)-1-phenylvinyl)phenyl)thiophene-3,4-diamine in $CDCl_3$ at 298 K.

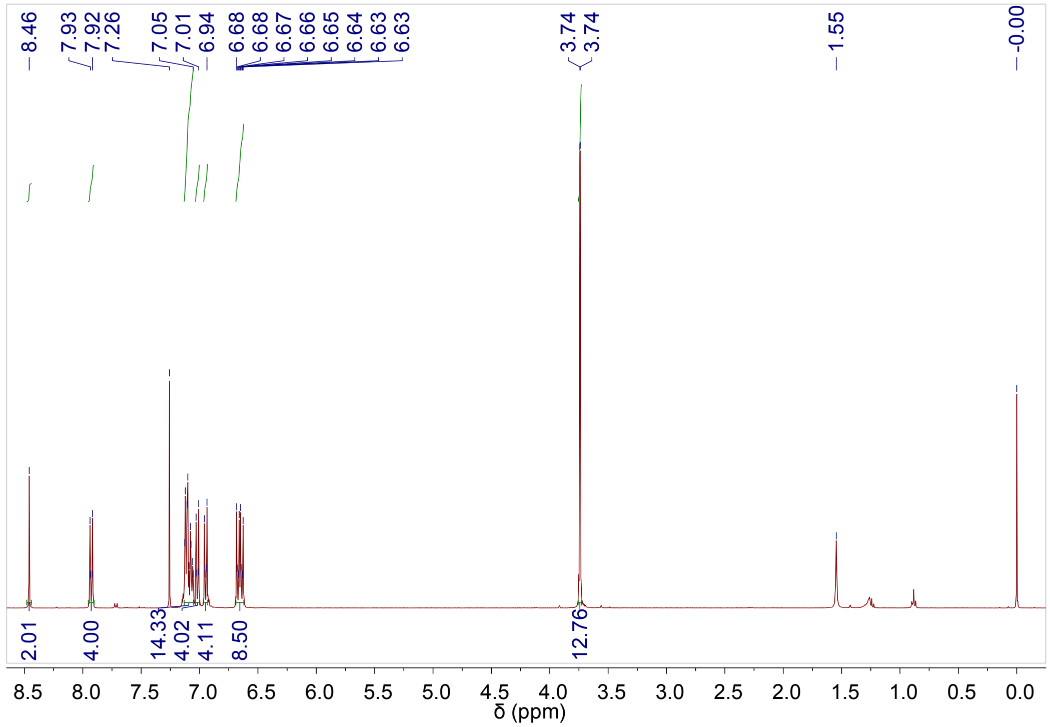

Fig. S10 ^{1}H NMR spectrum of MTPE-TP1 in $CDCl_3$ at 298 K.

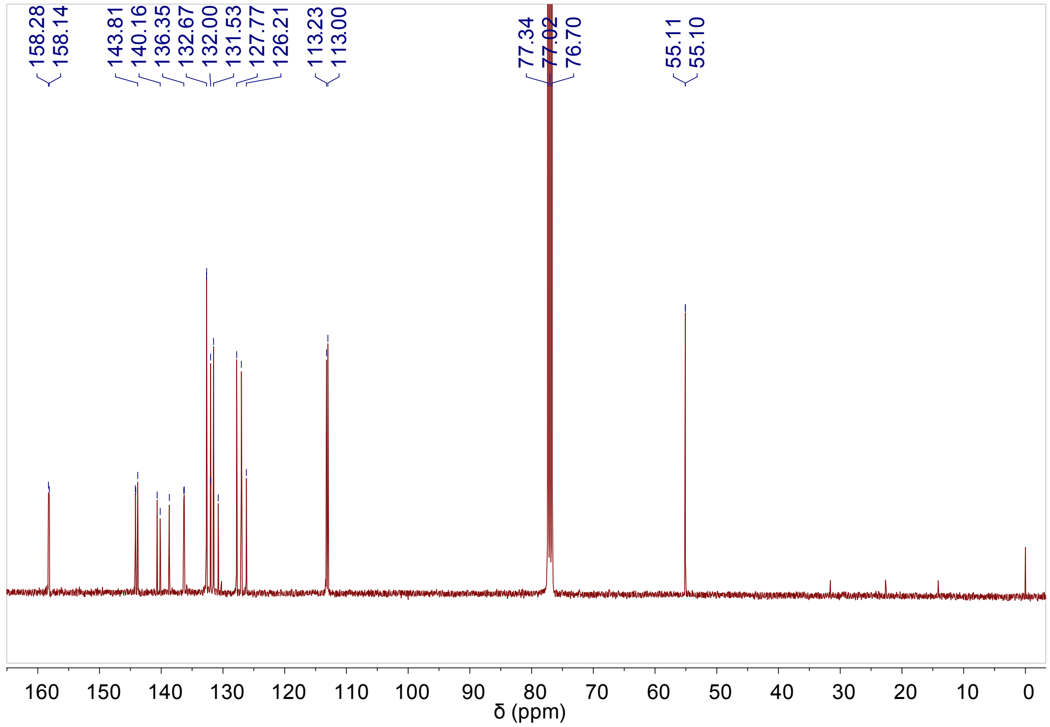

Fig. S11 ^{13}C NMR spectrum of MTPE-TP1 in $CDCl_3$ at 298 K.

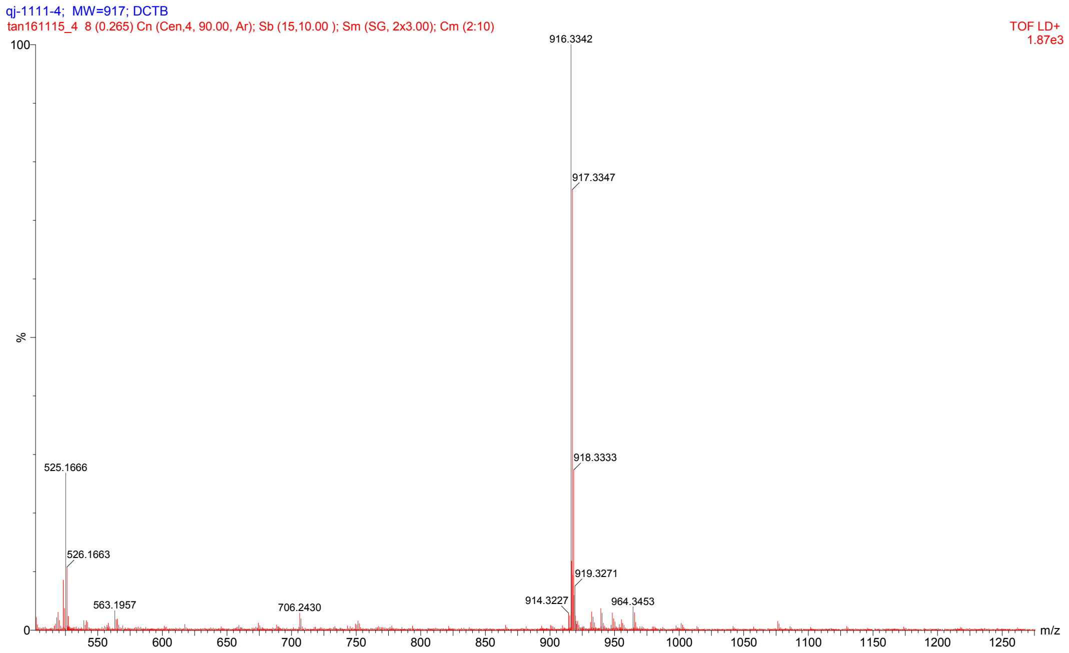

Fig. S12 HRMS of MTPE-TP1.

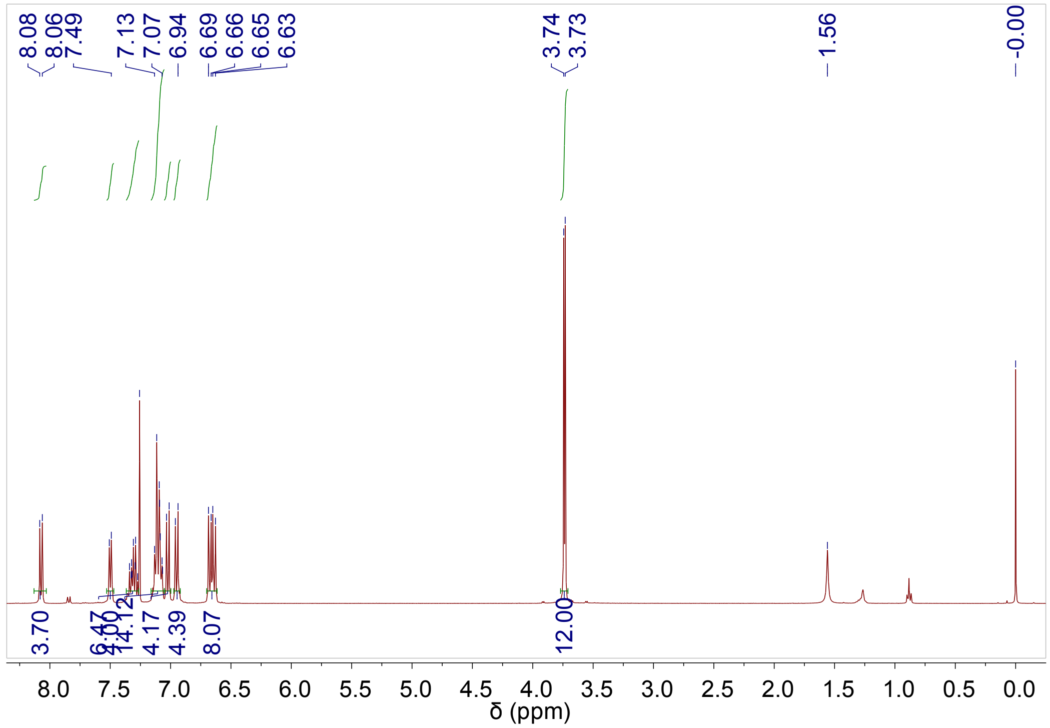

Fig. S13 ^{1}H NMR spectrum of MTPE-TP2 in $CDCl_3$ at 298 K.

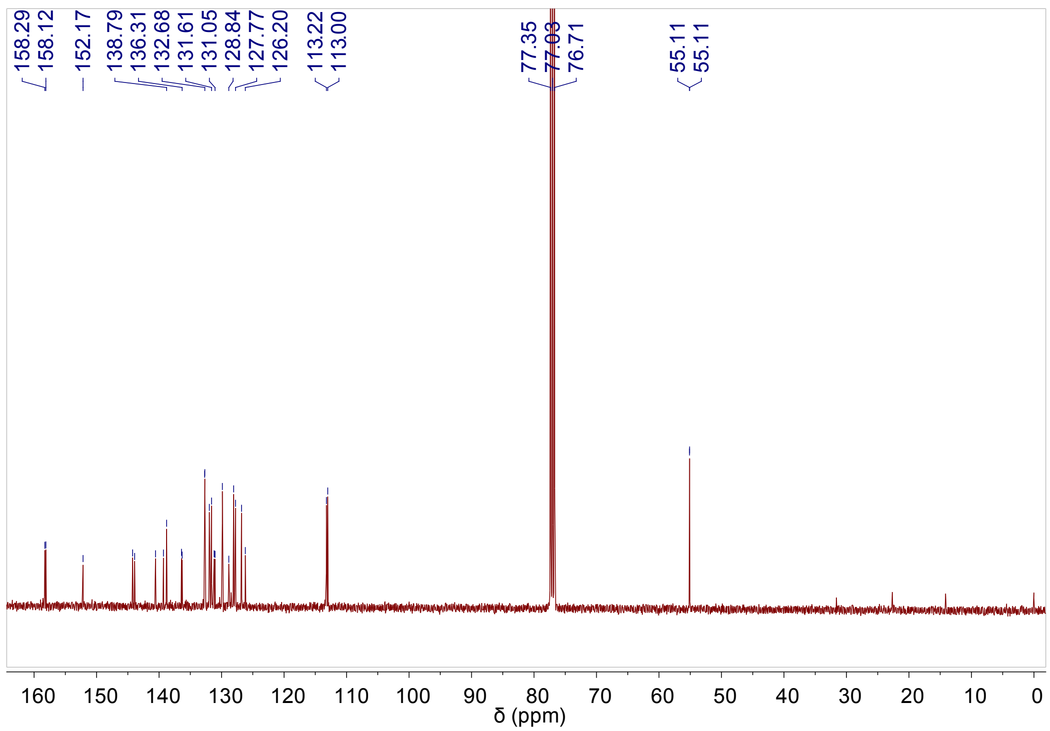

Fig. S14 ^{13}C NMR spectrum of MTPE-TP2 in $CDCl_3$ at 298 K.

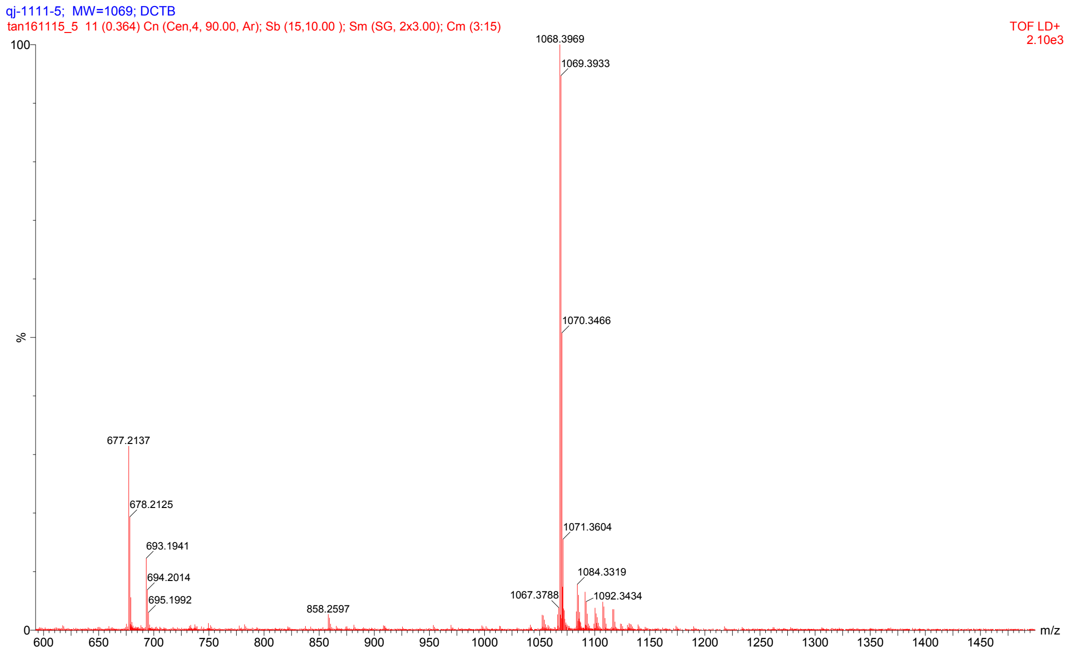

Fig. S15 HRMS of MTPE-TP2.

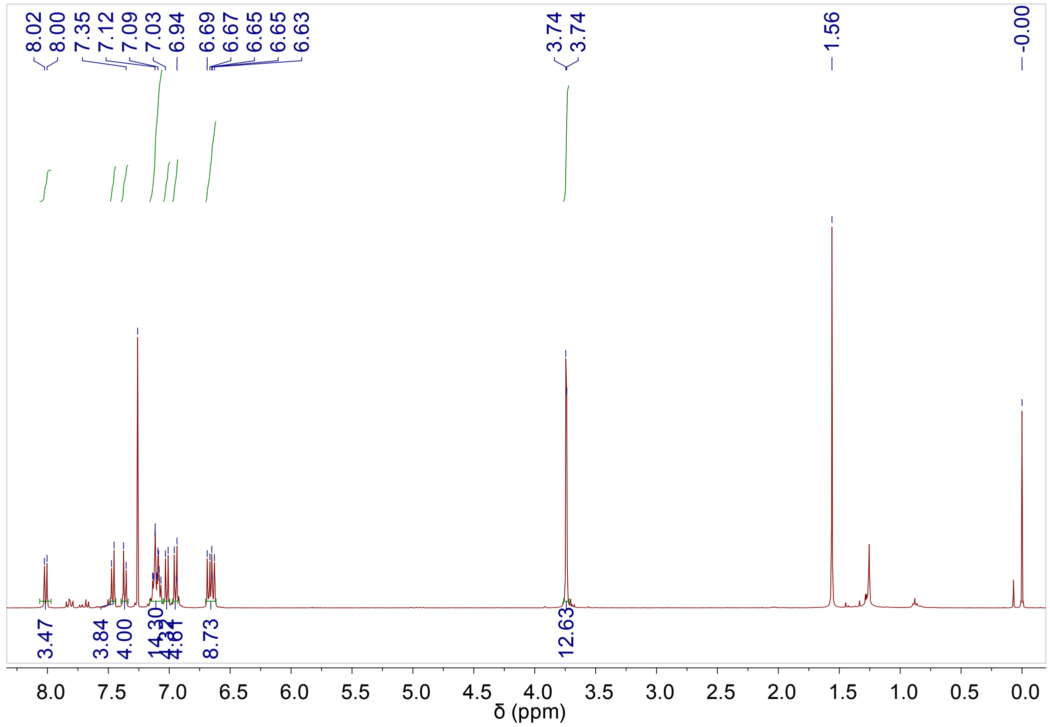

Fig. S16 ^{1}H NMR spectrum of 5,7-bis(4-(2,2-bis(4-methoxyphenyl)-1-phenylvinyl)phenyl)-2,3-bis(4-bromophenyl)thieno[3,4-*b*]pyrazine in $CDCl_3$ at 298 K.

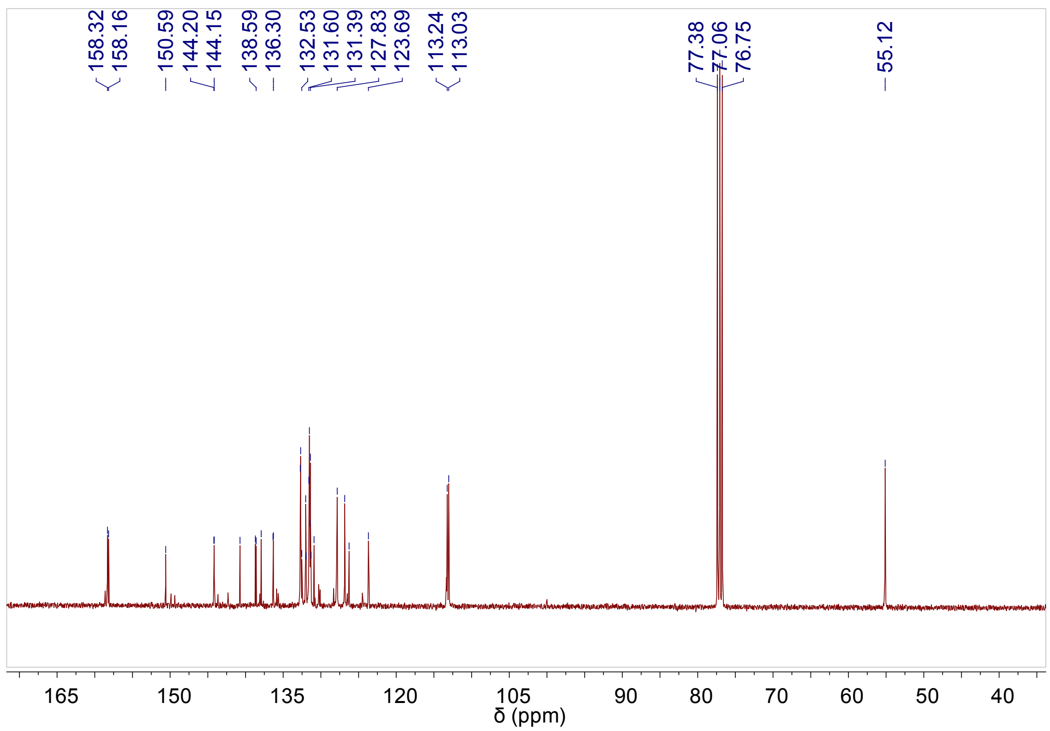

Fig. S17 ^{13}C NMR spectrum of 5,7-bis(4-(2,2-bis(4-methoxyphenyl)-1-phenylvinyl)phenyl)-2,3-bis(4-bromophenyl)thieno[3,4-*b*]pyrazine in $CDCl_3$ at 298 K.

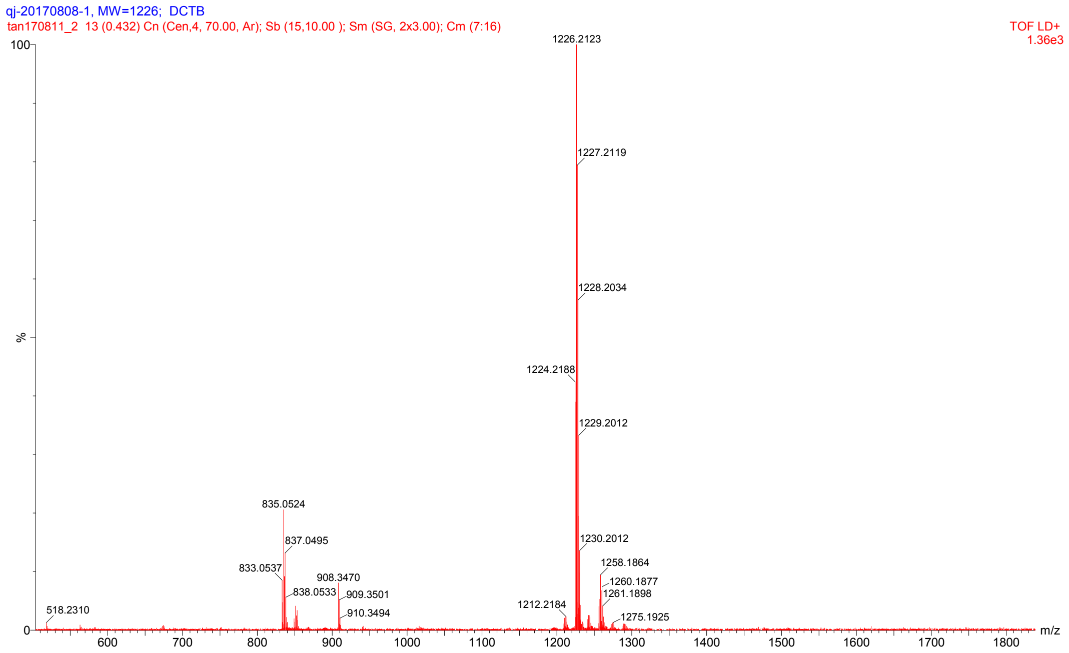

Fig. S18 HRMS of 5,7-bis(4-(2,2-bis(4-methoxyphenyl)-1-phenylvinyl)phenyl)-2,3-bis(4-bromophenyl)thieno[3,4-*b*]pyrazine.

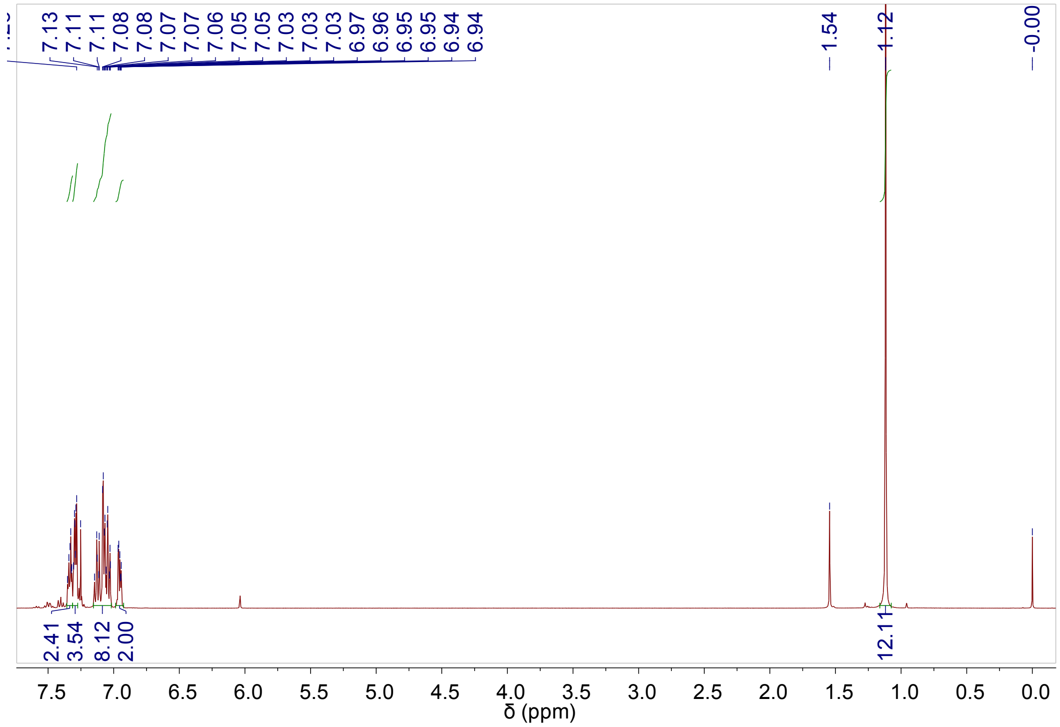

Fig. S19 ^{1}H NMR spectrum of 4,4,5,5-tetramethyl-2-(1,2,2-triphenylvinyl)-1,3,2-dioxaborolane in $CDCl_3$ at 298 K.

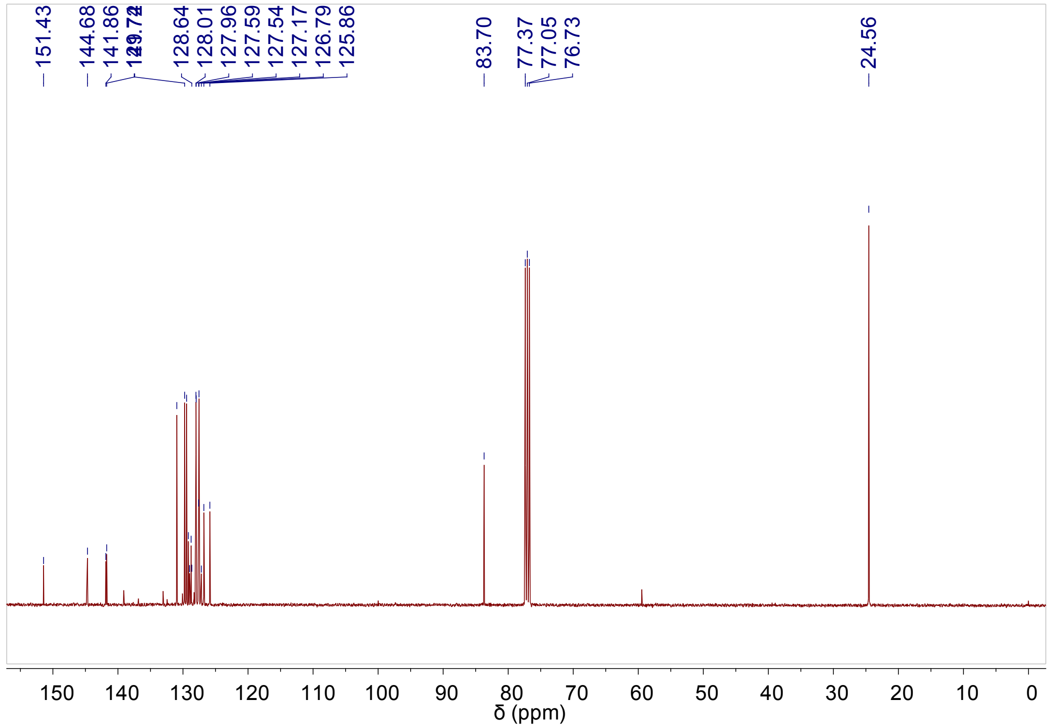

Fig. S20 ^{13}C NMR spectrum of 4,4,5,5-tetramethyl-2-(1,2,2-triphenylvinyl)-1,3,2-dioxaborolane in $CDCl_3$ at 298 K.

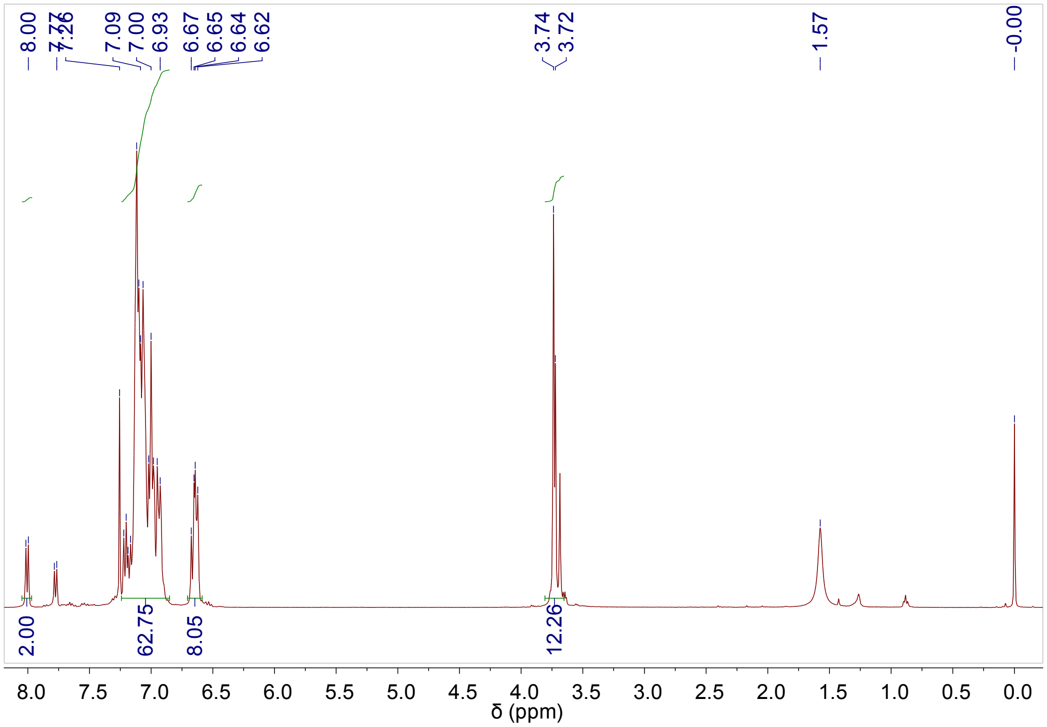

Fig. S21 ^{1}H NMR spectrum of MTPE-TP3 in $CDCl_3$ at 298 K.

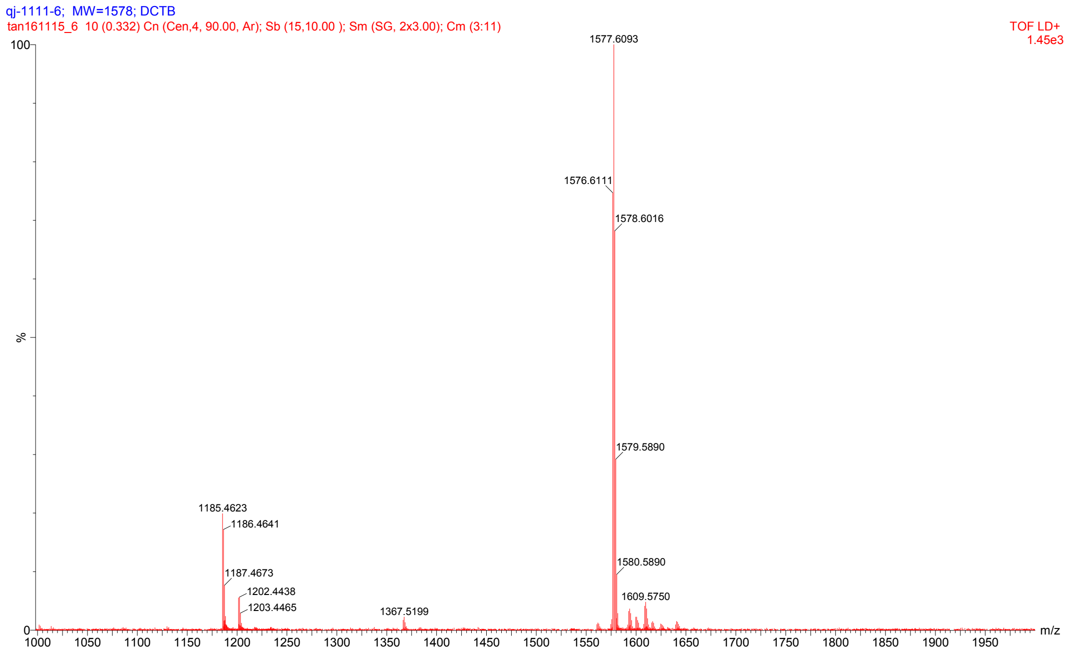

Fig. S22 HRMS of MTPE-TP3.

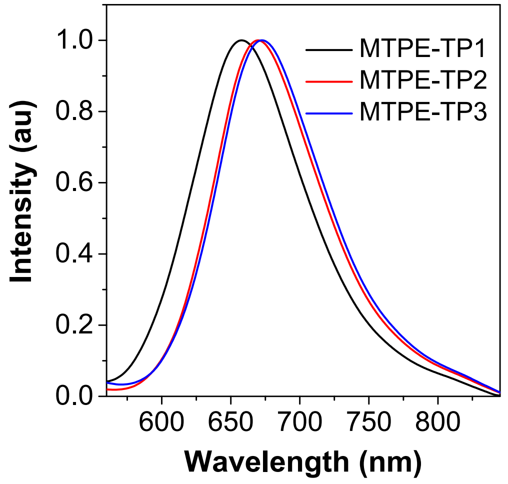

Fig. S23 PL spectra of MTPE-TP1-3 in DMSO (10^{-5} M).

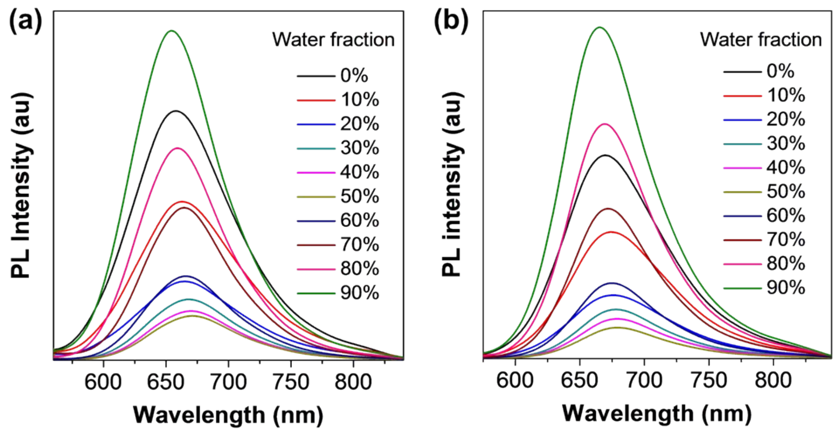

Fig. S24 PL spectra of (a) MTPE-TP1 and (b) MTPE-TP2 in DMSO/water mixtures with various water fractions.

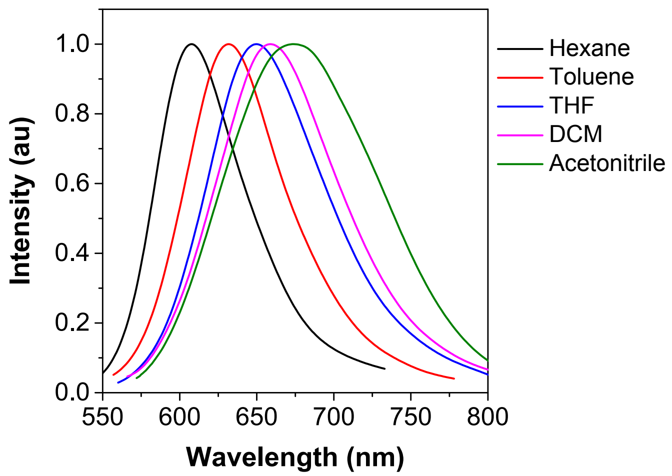

Fig. S25 PL spectra of MTPE-TP3 in different solvents (10^{-5} M).

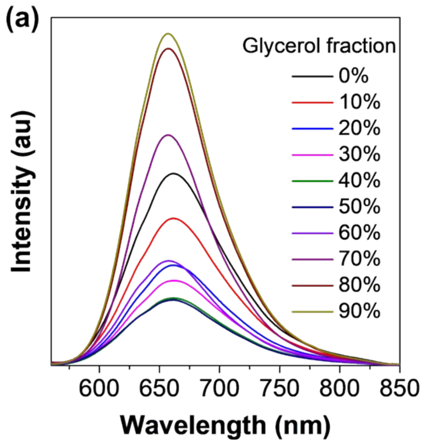

Fig. S26 PL spectra of (a) MTPE-TP1 and (b) MTPE-TP2 in DMF/glycerol mixtures with various glycerol fractions.

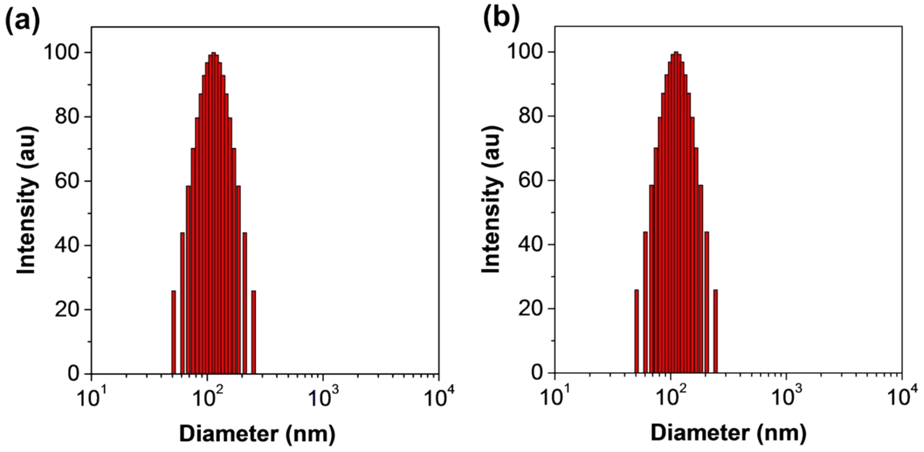

Fig. S27 Representative DLS results of (a) NPs1 and (b) NPs2.

Table S1. Comparison of PLQY of organic/polymer NIR NPs with similar NIR emission.

Reference	1	2	3	4	5	6	7	8	NPs3
λ_{PL} (nm)	650	655	655	670	672	680	682	700	685
Φ_F (%)	15.2	16.5	14.6	20	23	12	7	19	20.7

Table S2. Transient PL decay data for NPs1-3.

	τ_1 (ns)	A_1	τ_2 (ns)	A_2	τ_3 (ns)	A_3	R^2	τ^a (ns)
NPs1	1.96	1145	-	-	-	-	0.997	1.96
NPs2	2.26	1123	-	-	-	-	0.997	2.26
NPs3	3.11	601	0.96	617	12.9	13	0.997	3.25

[a] The fluorescence lifetime was calculated by $\tau = \Sigma A_i \tau_i^2 / \Sigma A_i \tau_i$, where A_i is the pre-exponential for lifetime τ_i.

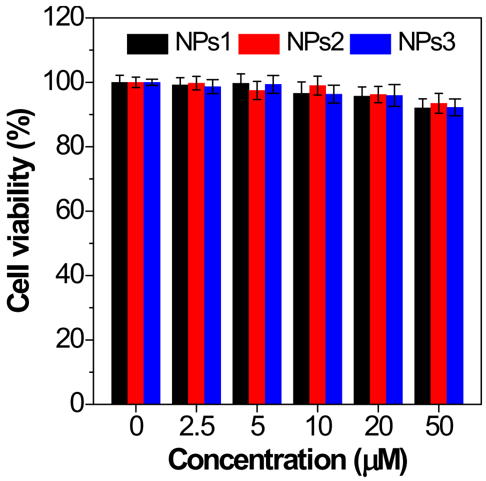

Fig. S28 Cell viabilities of 4T1 breast cancer cells incubated with NPs1-3 (n = 4).

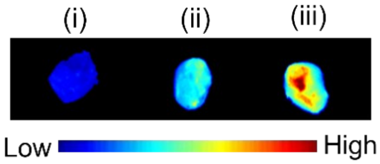

Fig. 29 Representative ex vivo fluorescence image of tumours after the injection of NPs1-3 into tumour-bearing mice for 24 h. (i)-(iii) correspond to NPs1-3, respectively.

Table S3. Cartesian coordinates of optimized MTPE-TP1 calculated by the DFT, B3LYP/6-31G(d), Gaussian 09 program.

atom	x	y	z
C	-14.16373	0.30056	-1.81078
O	-13.35533	-0.64267	-1.10957
C	-12.0234	-0.38152	-0.97998
C	-11.37742	0.75394	-1.48696
C	-10.00591	0.91398	-1.28223
C	-9.24199	-0.04341	-0.59879
C	-7.7819	0.15167	-0.38724
C	-6.87334	-0.85328	-0.57972
C	-5.46618	-0.76408	-0.10572
C	-4.40154	-1.16961	-0.93049
C	-3.08494	-1.10432	-0.49287
C	-2.76812	-0.63983	0.80071
C	-1.38259	-0.54854	1.25552
C	-0.85308	-0.56285	2.55162
C	0.59185	-0.44976	2.5909
C	1.18166	-0.34722	1.32579
S	-0.07225	-0.39401	0.12087
C	2.58745	-0.21773	0.94884
C	2.96635	0.38997	-0.26648
C	4.30269	0.49489	-0.6304
C	5.32405	-0.01404	0.19119
C	4.94675	-0.59925	1.41289
C	3.61312	-0.70575	1.78595
H	3.35313	-1.16282	2.73257
H	5.71691	-0.98569	2.07294
C	6.75356	0.11499	-0.20228
C	7.63426	-0.92726	-0.10787
C	9.10703	-0.7468	-0.22477
C	9.88149	-1.62173	-0.9999
C	11.26527	-1.47617	-1.11589
C	11.91095	-0.44175	-0.42601
C	11.15506	0.43094	0.37441
C	9.77999	0.27754	0.47016
H	9.20846	0.95959	1.09161
H	11.67079	1.21721	0.91747
O	13.25353	-0.20425	-0.45678
C	14.07335	-1.06553	-1.24457
H	13.79279	-1.0265	-2.30455
H	15.0935	-0.6972	-1.1261
H	14.01737	-2.10334	-0.89283
H	11.82143	-2.16859	-1.73677
H	9.39279	-2.43174	-1.53395
C	7.18112	-2.33069	0.11489

C	6.15465	-2.89409	-0.66257
C	5.7444	-4.21127	-0.45951
C	6.3543	-4.99358	0.52532
C	7.38627	-4.45074	1.29642
C	7.8024	-3.13655	1.08496
H	8.61016	-2.72036	1.68029
H	7.8691	-5.05242	2.06194
H	6.03364	-6.01938	0.68535
H	4.95118	-4.62891	-1.07395
H	5.67858	-2.2877	-1.42707
C	7.15081	1.45925	-0.70568
C	6.75917	2.6291	-0.02645
C	7.12079	3.88725	-0.48824
C	7.87517	4.01748	-1.66526
C	8.25494	2.86745	-2.371
C	7.89049	1.61065	-1.88701
H	8.19206	0.72554	-2.43845
H	8.8225	2.93707	-3.29149
O	8.17749	5.29424	-2.03751
C	8.94546	5.48749	-3.22383
H	8.42201	5.1007	-4.10715
H	9.07491	6.56628	-3.32316
H	9.92917	5.00774	-3.14643
H	6.82901	4.78672	0.04546
H	6.1704	2.54477	0.88259
H	4.56499	0.97822	-1.56688
H	2.20829	0.80488	-0.92543
N	1.26894	-0.42211	3.78127
C	0.53018	-0.5336	4.85892
C	-0.89745	-0.68092	4.81883
N	-1.58462	-0.69462	3.702
H	-1.44819	-0.78739	5.75152
H	1.03617	-0.51121	5.82228
C	-3.83746	-0.25609	1.63843
C	-5.15078	-0.32342	1.19247
H	-5.95408	-0.019	1.85573
H	-3.62675	0.08788	2.64326
H	-2.2941	-1.43757	-1.15961
H	-4.61344	-1.541	-1.92874
C	-7.22071	-2.11913	-1.28924
C	-6.87335	-3.36557	-0.74041
C	-7.18336	-4.55202	-1.40528
C	-7.83064	-4.51472	-2.64357
C	-8.16275	-3.2809	-3.21079
C	-7.85997	-2.09619	-2.54065
H	-8.12119	-1.13899	-2.98158

H	-8.65403	-3.24085	-4.17945
H	-8.06799	-5.43809	-3.16484
H	-6.91802	-5.50595	-0.95719
H	-6.3637	-3.39909	0.21868
C	-7.37494	1.51723	0.04359
C	-6.3061	2.19385	-0.57578
C	-5.93267	3.46929	-0.17826
C	-6.62116	4.11643	0.86104
C	-7.69852	3.46905	1.48105
C	-8.0675	2.19031	1.06022
H	-8.90803	1.70054	1.54362
H	-8.25305	3.94457	2.28155
O	-6.17299	5.36329	1.18255
C	-6.83475	6.06954	2.2305
H	-6.76861	5.52758	3.18209
H	-6.31557	7.02489	2.3206
H	-7.88954	6.24959	1.98806
H	-5.11206	3.99005	-0.66248
H	-5.76452	1.70538	-1.37983
C	-9.91586	-1.16992	-0.08706
C	-11.28001	-1.33992	-0.27153
H	-11.79692	-2.20571	0.13129
H	-9.35525	-1.91799	0.46426
H	-9.51706	1.80182	-1.67295
H	-11.92478	1.5127	-2.03363
H	-13.83722	0.41194	-2.85229
H	-15.17762	-0.10189	-1.79017
H	-14.15091	1.28164	-1.31975

Table S4. Cartesian coordinates of optimized MTPE-TP2 calculated by the DFT, B3LYP/6-31G(d), Gaussian 09 program.

atom	x	y	z
C	14.36839	-2.30274	0.24308
O	13.4987	-1.69081	-0.70769
C	12.16805	-1.62566	-0.41722
C	11.5801	-2.11729	0.75586
C	10.20316	-1.98468	0.94287
C	9.37793	-1.38993	-0.02275
C	7.9125	-1.25728	0.20058
C	6.99443	-1.55244	-0.77047
C	5.56265	-1.16445	-0.66356
C	4.54283	-2.07247	-1.0006
C	3.20287	-1.71929	-0.90756
C	2.81366	-0.43125	-0.48328
C	1.40534	-0.06619	-0.35873
C	0.79492	1.19367	-0.31151

C	-0.63852	1.13593	-0.1752
C	-1.14705	-0.16103	-0.0826
S	0.16772	-1.29047	-0.18851
C	-2.54346	-0.56036	0.04524
C	-3.06427	-1.69312	-0.6091
C	-4.42205	-1.98888	-0.54567
C	-5.31522	-1.15699	0.15317
C	-4.77758	-0.07134	0.86787
C	-3.42462	0.22557	0.8163
H	-3.03658	1.07249	1.36985
H	-5.43959	0.55613	1.45526
C	-6.7804	-1.41369	0.13283
C	-7.68096	-0.40273	-0.07201
C	-9.14083	-0.56191	0.16417
C	-10.07735	-0.05481	-0.74832
C	-11.45235	-0.18399	-0.54288
C	-11.92059	-0.81867	0.61503
C	-10.99812	-1.31046	1.55372
C	-9.6358	-1.18232	1.32851
H	-8.93476	-1.56639	2.06261
H	-11.37542	-1.78293	2.45567
O	-13.23587	-0.99971	0.9265
C	-14.21847	-0.51864	0.01129
H	-14.12781	-1.00748	-0.96677
H	-15.18474	-0.76745	0.45267
H	-14.14621	0.5684	-0.11871
H	-12.13908	0.21152	-1.28191
H	-9.72601	0.4456	-1.64625
C	-7.25251	0.94735	-0.54501
C	-6.40854	1.09265	-1.65965
C	-5.97927	2.35288	-2.07319
C	-6.39121	3.49717	-1.38417
C	-7.25322	3.36973	-0.291
C	-7.68857	2.10838	0.11636
H	-8.35676	2.01337	0.96783
H	-7.58374	4.25406	0.24767
H	-6.04331	4.47759	-1.69569
H	-5.32039	2.44234	-2.93283
H	-6.07664	0.2047	-2.189
C	-7.1875	-2.83154	0.33022
C	-6.61431	-3.60994	1.35427
C	-6.98253	-4.93464	1.54599
C	-7.92953	-5.53374	0.70001
C	-8.49434	-4.78502	-0.34183
C	-8.11889	-3.45242	-0.51418
H	-8.56326	-2.87947	-1.32203

H	-9.21449	-5.22529	-1.0213
O	-8.22345	-6.83812	0.96912
C	-9.1859	-7.49664	0.14788
H	-8.85876	-7.53782	-0.89859
H	-9.26985	-8.51089	0.54114
H	-10.16382	-7.00211	0.20274
H	-6.54919	-5.52819	2.34532
H	-5.8764	-3.16123	2.0133
H	-4.80758	-2.85181	-1.08067
H	-2.40768	-2.31797	-1.20881
N	-1.39644	2.26361	-0.2533
C	-0.77635	3.4184	-0.36943
C	0.69025	3.49131	-0.33843
N	1.42902	2.40071	-0.35636
C	1.43451	4.77435	-0.24259
C	1.01714	5.79741	0.62455
C	1.76347	6.96888	0.74491
C	2.93	7.13982	-0.00566
C	3.35454	6.12529	-0.86852
C	2.61698	4.94741	-0.98007
H	2.945	4.15306	-1.6431
H	4.26031	6.25157	-1.45508
H	3.50543	8.05711	0.08277
H	1.43404	7.74808	1.42648
H	0.11288	5.67124	1.21121
C	-1.6587	4.5947	-0.59737
C	-1.32919	5.60354	-1.51808
C	-2.2159	6.6507	-1.76679
C	-3.4403	6.71185	-1.09563
C	-3.77924	5.70967	-0.18185
C	-2.89962	4.6556	0.05881
H	-3.16718	3.86665	0.75424
H	-4.73116	5.74484	0.34024
H	-4.12659	7.53235	-1.28576
H	-1.95077	7.4178	-2.48895
H	-0.38444	5.5621	-2.04969
C	3.83779	0.49173	-0.17512
C	5.17459	0.12876	-0.26832
H	5.9411	0.85613	-0.02026
H	3.57092	1.49601	0.1293
H	2.44917	-2.45175	-1.18445
H	4.80821	-3.07035	-1.33714
C	7.35626	-2.29917	-2.01062
C	6.94103	-1.83703	-3.27136
C	7.26496	-2.53808	-4.43308
C	7.99424	-3.72792	-4.35528

C	8.39517	-4.21005	-3.1058
C	8.0781	-3.50334	-1.94637
H	8.39274	-3.8778	-0.97703
H	8.95156	-5.14089	-3.03349
H	8.24236	-4.27756	-5.25917
H	6.94588	-2.15636	-5.39936
H	6.3671	-0.91662	-3.33587
C	7.5127	-0.78859	1.55584
C	6.49866	-1.4372	2.28734
C	6.13266	-1.00407	3.55321
C	6.77383	0.10177	4.1355
C	7.79695	0.75292	3.43319
C	8.15956	0.29623	2.16488
H	8.95823	0.80463	1.6322
H	8.31451	1.60492	3.85816
O	6.33735	0.45288	5.37856
C	6.9534	1.56767	6.02085
H	6.81751	2.48906	5.44098
H	6.45398	1.66963	6.98551
H	8.02491	1.39513	6.18178
H	5.3546	-1.51061	4.11633
H	5.99364	-2.29229	1.84912
C	9.99361	-0.89145	-1.18776
C	11.36184	-1.00462	-1.38542
H	11.83349	-0.61239	-2.28141
H	9.38353	-0.40902	-1.94485
H	9.75933	-2.36299	1.85926
H	12.17641	-2.597	1.52303
H	14.1054	-3.3552	0.40732
H	15.36962	-2.24236	-0.18619
H	14.3503	-1.77052	1.20237

Table S5. Cartesian coordinates of optimized MTPE-TP3 calculated by the DFT, B3LYP/6-31G(d), Gaussian 09 program.

atom	x	y	z
C	-1.49026	-3.56997	0.11065
C	-0.83567	-2.34135	0.02517
C	0.59216	-2.45546	-0.12976
C	1.04317	-3.77423	-0.18633
S	-0.31891	-4.85105	-0.02835
N	1.3892	-1.34651	-0.10324
N	-1.44953	-1.12295	-0.01216
C	2.42814	-4.22871	-0.26767
C	2.90453	-5.31092	0.49464
C	3.34553	-3.53715	-1.08506
C	4.2541	-5.64642	0.48566

H	2.22036	-5.86123	1.13503
C	4.69016	-3.87777	-1.08941
H	2.9919	-2.72755	-1.71325
C	5.18465	-4.91171	-0.2728
H	4.60247	-6.46736	1.10489
H	5.37787	-3.32624	-1.72153
C	6.63463	-5.23476	-0.20792
C	7.60945	-4.27552	-0.14949
C	6.95693	-6.6926	-0.18313
C	9.05151	-4.60783	-0.32227
C	7.32016	-2.83104	0.07781
C	6.39976	-7.56167	-1.13648
C	7.78399	-7.23705	0.81351
C	10.01742	-4.08643	0.55044
C	9.50089	-5.41833	-1.38275
C	6.43186	-2.38955	1.0784
C	7.95307	-1.85222	-0.70256
C	6.68698	-8.92659	-1.11518
H	5.74351	-7.15735	-1.90244
C	8.06478	-8.60278	0.84216
H	8.2102	-6.57738	1.56319
C	11.37448	-4.38115	0.4095
H	9.70194	-3.44254	1.36648
C	10.84705	-5.7116	-1.54563
H	8.77834	-5.82137	-2.0852
C	6.16664	-1.0398	1.26495
H	5.93826	-3.12125	1.71025
C	7.69506	-0.49136	-0.53274
H	8.65719	-2.15951	-1.47029
C	7.52205	-9.45298	-0.12567
H	6.25716	-9.57957	-1.8702
H	8.70462	-9.00443	1.62353
C	11.79666	-5.20172	-0.64509
H	12.08382	-3.96848	1.11704
H	11.19047	-6.33126	-2.36853
C	6.79149	-0.07801	0.45443
H	5.48039	-0.70463	2.03682
H	8.20016	0.22718	-1.16715
H	7.74258	-10.51676	-0.10504
C	-2.93035	-3.78171	0.22799
C	-3.61247	-4.76148	-0.51643
C	-3.68537	-2.93317	1.0627
C	-5.00181	-4.83656	-0.48437
H	-3.05293	-5.43158	-1.16379
C	-5.06937	-3.01165	1.08674
H	-3.17458	-2.20527	1.68255

C	-5.76127	-3.92928	0.27631
H	-5.51213	-5.57708	-1.09319
H	-5.63249	-2.3358	1.72174
C	-7.2481	-3.93133	0.21454
C	-7.96163	-2.7693	0.09121
C	-7.89208	-5.27191	0.27474
C	-9.43594	-2.707	0.28471
C	-7.31004	-1.46576	-0.23612
C	-7.47954	-6.22593	1.22505
C	-8.89526	-5.64832	-0.6297
C	-10.24943	-1.99273	-0.6064
C	-10.06055	-3.32106	1.38814
C	-6.40878	-1.3455	-1.30839
C	-7.59614	-0.31859	0.52468
C	-8.06692	-7.48208	1.2892
H	-6.69149	-5.97047	1.92786
C	-9.49144	-6.9089	-0.58505
H	-9.22035	-4.93813	-1.38354
C	-11.63344	-1.91336	-0.43982
H	-9.79384	-1.49299	-1.4567
C	-11.43264	-3.24313	1.57546
H	-9.45391	-3.86601	2.10448
C	-5.78229	-0.13063	-1.58425
H	-6.18924	-2.21984	-1.91353
C	-6.96566	0.89605	0.25401
H	-8.30482	-0.39072	1.34501
C	-9.08177	-7.83405	0.38513
H	-7.75545	-8.21054	2.03174
H	-10.25991	-7.15799	-1.30741
C	-12.23409	-2.54419	0.65752
H	-12.22403	-1.36182	-1.16186
H	-11.90934	-3.71167	2.43123
C	-6.05215	0.99394	-0.79914
H	-5.08214	-0.06051	-2.4125
H	-7.18477	1.76624	0.86732
H	-5.55481	1.93701	-1.00384
O	13.09155	-5.55445	-0.88678
O	6.46501	1.2226	0.7068
O	-9.59352	-9.09013	0.52818
O	-13.57094	-2.53215	0.92706
C	14.10036	-5.06666	-0.00415
H	13.93538	-5.4146	1.02322
H	15.04318	-5.4702	-0.37651
H	14.14443	-3.97037	-0.01302
C	7.08016	2.23922	-0.08145
H	6.87184	2.10028	-1.14917

H	6.63527	3.17756	0.24735
H	8.16503	2.26511	0.07738
C	-10.62919	-9.50242	-0.36164
H	-10.28566	-9.50222	-1.4037
H	-10.88899	-10.52007	-0.06618
H	-11.51368	-8.85951	-0.27152
C	-14.43371	-1.83549	0.02999
H	-14.38806	-2.26062	-0.98045
H	-15.44158	-1.95786	0.42953
H	-14.18674	-0.76736	-0.01478
C	-0.6752	-0.06326	-0.08334
C	0.78837	-0.17949	-0.03133
C	1.65331	1.01025	0.15919
C	1.35346	1.96662	1.14323
C	2.80841	1.178	-0.61808
C	2.18518	3.06261	1.33525
H	0.46745	1.85046	1.75953
C	3.60984	2.30282	-0.45618
H	3.06018	0.42934	-1.36301
C	3.30597	3.26962	0.51516
H	1.95786	3.78336	2.11483
H	4.47422	2.44518	-1.09406
C	4.14878	4.48434	0.70373
C	4.44867	5.3175	-0.33247
C	4.5968	4.71875	2.10634
C	5.42646	6.4387	-0.23067
C	3.7886	5.17013	-1.66461
C	5.19621	3.67857	2.83805
C	4.38914	5.95577	2.73834
C	5.12009	7.68845	-0.79862
C	6.6794	6.27998	0.38716
C	2.38864	5.14339	-1.78064
C	4.561	5.09004	-2.83487
C	5.60832	3.8844	4.15485
H	5.35254	2.71299	2.36475
C	4.78929	6.15612	4.05909
H	3.91334	6.75913	2.18404
C	6.02175	8.75002	-0.72677
H	4.16292	7.82575	-1.29331
C	7.58631	7.33677	0.44955
H	6.94095	5.32389	0.8273
C	1.78027	5.02012	-3.02952
H	1.77978	5.21441	-0.88431
C	3.95346	4.95132	-4.08289
H	5.64431	5.12659	-2.75838
C	5.40616	5.12289	4.77062

H	6.08468	3.07519	4.70209
H	4.61619	7.11804	4.53425
C	7.2601	8.57876	-0.10252
H	5.75785	9.71042	-1.16145
H	8.55138	7.18829	0.92657
C	2.55982	4.91594	-4.18477
H	0.69626	5.01394	-3.10272
H	4.56761	4.87391	-4.97604
H	5.72072	5.27991	5.79877
H	7.96663	9.40265	-0.05213
H	2.0851	4.81524	-5.1569
C	-1.35046	1.24543	-0.25948
C	-0.88299	2.18844	-1.19085
C	-2.50545	1.54144	0.47946
C	-1.54322	3.39898	-1.35967
H	3.6599E-4	1.97404	-1.7837
C	-3.13738	2.77218	0.33829
H	-2.89343	0.80362	1.17463
C	-2.65671	3.72869	-0.57027
H	-1.18257	4.11279	-2.094
H	-4.01003	3.00487	0.9403
C	-3.32221	5.05338	-0.71094
C	-3.54087	5.85405	0.37199
C	-3.6999	5.42866	-2.10216
C	-4.43054	7.04797	0.32492
C	-2.89451	5.58684	1.69031
C	-4.31099	4.49116	-2.95302
C	-3.40291	6.70287	-2.61674
C	-4.02034	8.26895	0.88738
C	-5.71394	6.97005	-0.24286
C	-1.50939	5.36401	1.78466
C	-3.65276	5.59484	2.87321
C	-4.64716	4.8273	-4.26436
H	-4.52623	3.49388	-2.57969
C	-3.72912	7.03662	-3.93027
H	-2.91293	7.42995	-1.97674
C	-4.85307	9.38738	0.85099
H	-3.03799	8.33854	1.34612
C	-6.55116	8.08441	-0.27021
H	-6.04831	6.02693	-0.66418
C	-0.90662	5.13246	3.02034
H	-0.90788	5.37055	0.88076
C	-3.05256	5.34981	4.10832
H	-4.7211	5.78462	2.81721
C	-4.35832	6.1022	-4.75829
H	-5.13137	4.09201	-4.90139

H	-3.48608	8.02538	-4.31016
C	-6.12159	9.29987	0.2707
H	-4.51251	10.3268	1.27819
H	-7.54179	8.0029	-0.70958
C	-1.67653	5.11684	4.18682
H	0.16684	4.97325	3.07429
H	-3.65873	5.34356	5.01026
H	-4.61365	6.3629	-5.78172
H	-6.77315	10.16909	0.24754
H	-1.20698	4.9327	5.14919

References

1. F. Zhang, Y. Di, Y. Li, Q. Qi, J. Qian, X. Fu, B. Xu and W. Tian, *Dyes Pigments*, 2017, **142**, 491.

2. X. Cai, A. Bandla, C. K. Chuan, G. Magarajah, L.-D. Liao, D. B. L. Teh, B. K. Kennedy, N. V. Thakor and B. Liu, *Mater. Horiz.*, 2019, **6**, 311.

3. L. Meng, X. Ma, S. Jianga, G. Jia, W. Hana, B. Xu, J. Tian and W. Tian, *J. Mater. Chem. B*, 2019, **7**, 5345.

4. C. Teh, P. N. Manghnani, G. N. H. Boon, T. Y. D. Cheng, W.-T. Lim, E. H. Lim, B. T. Chua and B. Liu, *Adv. Funct. Mater.*, 2019, **29**, 1901226.

5. Z. Zhang, D. Chen, Z. Liu, D. Wang, J. Guo, J. Zheng, W. Qin and C. Wu, *ACS Appl. Polym. Mater.*, 2020, **2**, 74.

6. A. Nicol, W. Qin, R. T. K. Kwok, J. M. Burkhartsmeyer, Z. Zhu, H. Su, W. Luo, J. W. Y. Lam, J. Qian, K. S. Wong and B. Z. Tang, *Chem. Sci.*, 2017, **8**, 4634.

7. P. Koralli, A. D. Nega, L. E. Vagiaki, A. Pavlou, M. G. Siskos, A. Dimitrakopoulou-Strauss, V. G. Gregoriou and C. L. Chochos, *Mater. Chem. Front.*, 2020, DOI: 10.1039/D0QM00195C.

8. S. Wang, J. Liu, C. C. Goh, L. G. Ng and B. Liu, *Adv. Mater.*, 2019, **31**, 1904447.
